# Mosquitoes provide a transmission route between possums and humans for Buruli ulcer in southeastern Australia

**DOI:** 10.1038/s41564-023-01553-1

**Published:** 2024-01-23

**Authors:** Peter T. Mee, Andrew H. Buultjens, Jane Oliver, Karen Brown, Jodie C. Crowder, Jessica L. Porter, Emma C. Hobbs, Louise M. Judd, George Taiaroa, Natsuda Puttharak, Deborah A. Williamson, Kim R. Blasdell, Ee Laine Tay, Rebecca Feldman, Mutizwa Odwell Muzari, Chris Sanders, Stuart Larsen, Simon R. Crouch, Paul D. R. Johnson, John R. Wallace, David J. Price, Ary A. Hoffmann, Katherine B. Gibney, Timothy P. Stinear, Stacey E. Lynch

**Affiliations:** 1 Centre for AgriBioscience, AgriBio, Agriculture Victoria, Bundoora, Victoria Australia; 2 Department of Microbiology and Immunology, Doherty Institute for Infection and Immunity, University of Melbourne, Melbourne, Victoria Australia; 3 Department of Infectious Diseases, Doherty Institute for Infection and Immunity, University of Melbourne, Melbourne, Victoria Australia; 4 Victorian Infectious Diseases Reference Laboratory, Doherty Institute for Infection and Immunity, Melbourne Health, Melbourne, Victoria Australia; 5 Australian Centre for Disease Preparedness, CSIRO, Geelong, Victoria Australia; 6 Department of Health, Melbourne, Victoria Australia; 7 Medical Entomology, Tropical Public Health Services Cairns, Cairns and Hinterland Hospital and Health Services, Cairns, Queensland Australia; 8 South East Public Health Unit, Monash Health, Clayton, Victoria Australia; 9 North East Public Health Unit, Austin Health, Heidelberg, Victoria Australia; 10 Department of Biology, Millersville University, Millersville, PA USA; 11 Centre for Epidemiology and Biostatistics, Melbourne School of Population and Global Health, University of Melbourne, Parkville, Victoria Australia; 12 Pest and Environmental Adaptation Research Group, School of BioSciences, Bio21 Institute, University of Melbourne, Parkville, Victoria Australia; 13 WHO Collaborating Centre for Mycobacterium ulcerans, Doherty Institute for Infection and Immunity, Melbourne, Victoria Australia

**Keywords:** Bacteriology, Bacterial infection

## Abstract

Buruli ulcer, a chronic subcutaneous infection caused by *Mycobacterium ulcerans*, is increasing in prevalence in southeastern Australia. Possums are a local wildlife reservoir for *M. ulcerans* and, although mosquitoes have been implicated in transmission, it remains unclear how humans acquire infection. We conducted extensive field survey analyses of *M. ulcerans* prevalence among mosquitoes in the Mornington Peninsula region of southeastern Australia. PCR screening of trapped mosquitoes revealed a significant association between *M. ulcerans* and *Aedes notoscriptus*. Spatial scanning statistics revealed overlap between clusters of *M. ulcerans*-positive *Ae. notoscriptus*, *M. ulcerans*-positive possum excreta and Buruli ulcer cases, and metabarcoding analyses showed individual mosquitoes had fed on humans and possums. Bacterial genomic analysis confirmed shared single-nucleotide-polymorphism profiles for *M. ulcerans* detected in mosquitoes, possum excreta and humans. These findings indicate *Ae. notoscriptus* probably transmit *M. ulcerans* in southeastern Australia and highlight mosquito control as a Buruli ulcer prevention measure.

## Abstract (web summary)

Genomic evidence reveals that *Mycobacterium ulcerans* recovered from *Aedes notoscriptus* mosquitoes are genetically identical to bacteria from possums and humans, implicating mosquitoes as a vector for Buruli’s ulcer transmission in southeastern Australia.

## Main

**Extended Data Fig. 1:**
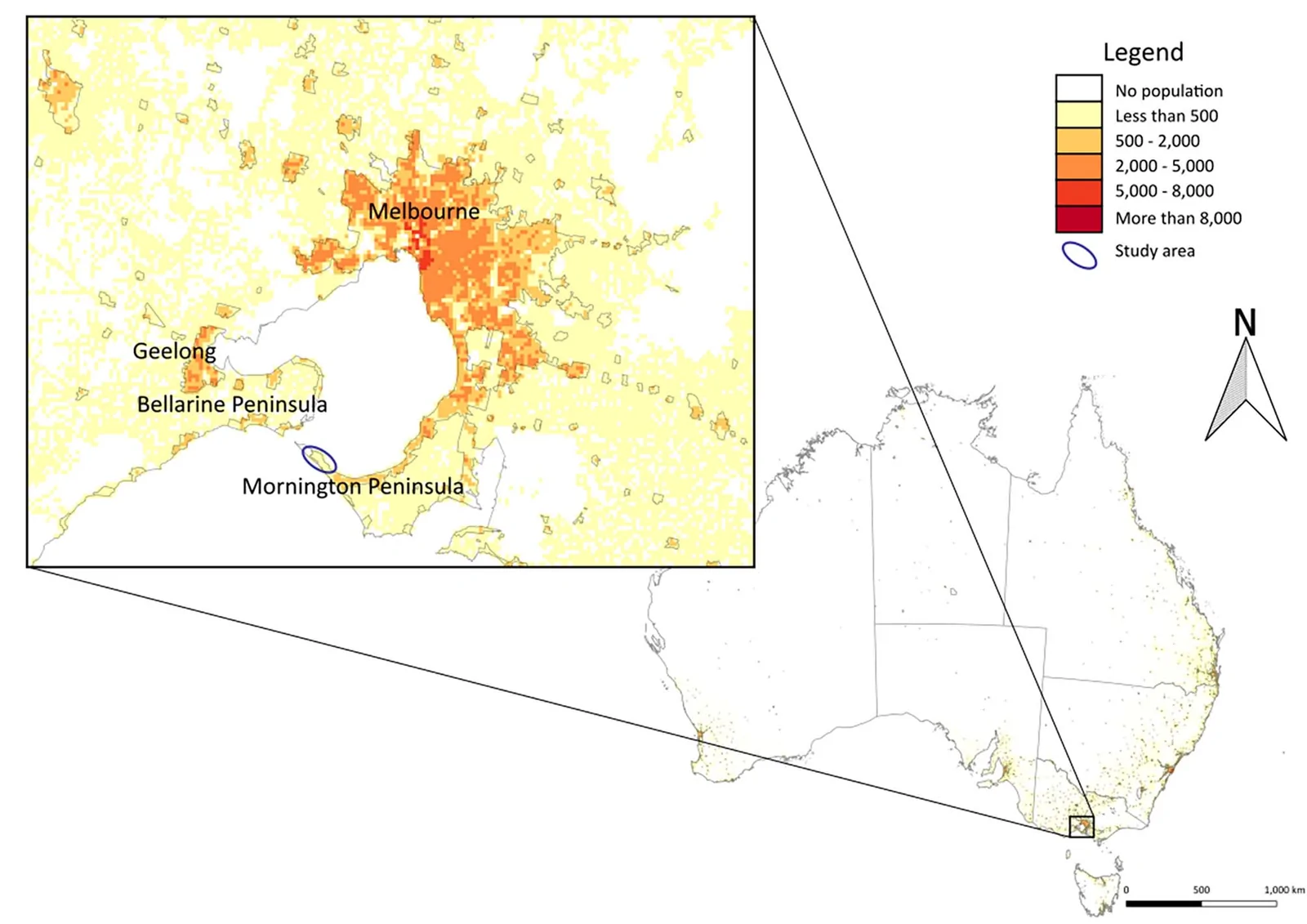
Location of the Mornington Peninsula and the Bellarine Peninsula of Victoria, Australia. Population density is represented on the map from low to high (white to red) based on the human population per square kilometre. The study area is shown within an elipse (inset). Map boundaries in **a** from Australian Bureau of Statistics^48^ under a Creative Commons license CC BY 4.0.

*Mycobacterium ulcerans* is the causative agent of a neglected tropical skin disease called Buruli ulcer, a necrotizing infection of skin and subcutaneous tissue^1^. Buruli ulcer is rarely a fatal condition but it can cause severe tissue destruction if not diagnosed and managed effectively^2^. Buruli ulcer has been described in more than 32 countries worldwide^3^ and is an ongoing public health issue in West and Central Africa^2^. Buruli ulcer has also been unexpectedly surging in temperate southeastern Australia (Extended Data Fig. 1) and encroaching on the major metropolitan centres of Melbourne (population 5.1 million) and Geelong (population 274,000), with more than 250 cases routinely notified each year since 2017 to the Victorian state government Department of Health^4^.

How humans contract Buruli ulcer is a central question that has confounded public health control efforts and intrigued scientists since the discovery of *M. ulcerans* from patients in the Bairnsdale region of Australia in the 1930s and across Africa shortly thereafter^1,5,6^. Buruli ulcer epidemiology can be unpredictable, with a 4–5 month median incubation period and outbreaks emerging in specific geographical areas and then disappearing over a number of years. It is also very challenging to isolate the bacterium in pure culture from the environment, presumably due to its very slow growth, although it can be isolated from human skin lesions. These factors combined have made it incredibly challenging to establish how *M. ulcerans* is spread to humans, despite global research efforts over more than 80 years^7^.

The discovery that Buruli ulcer is a zoonosis and that Australian native possums are a major wildlife source of *M. ulcerans* that is intimately linked with disease transmission has addressed one key component of the transmission enigma^8–13^. The first indications that mosquitoes might be vectors of *M. ulcerans* from possums to humans in Australia came from a series of entomological field surveys in the southeast of the country in response to an increase in Buruli ulcer cases in the seaside township of Point Lonsdale, located on the Bellarine Peninsula^14^. Among the 12 species identified from a trapping effort that collected 11,500 mosquitoes, 5 different species were IS*2404* PCR positive for *M. ulcerans*, including *Aedes camptorhynchus*, *Aedes notoscriptus*, *Coquillettidia linealis*, *Culex australicus* and *Anopheles annulipes* (maximum likelihood estimate (MLE) was 4.11 per 1,000 mosquitoes)^14^. IS*2404* is a *M. ulcerans*-specific insertion sequence and molecular target for the gold-standard diagnostic PCR for Buruli ulcer^15^.

A concurrent case–control study performed in the same geographical area identified only two factors associated with the odds of being diagnosed with Buruli ulcer: insect repellent use reduced risk (odds ratio (OR), 0.37; 95% confidence interval (CI), 0.19–0.69) and being bitten by mosquitoes on the lower legs increased risk (OR, 2.60; 95% CI, 1.22–5.53). A variety of outdoor activities was also surveyed but was not independently predictive, suggesting that mosquito exposure specifically rather than environmental exposure generally might be the main mode of *M. ulcerans* transmission to humans^16^. In Africa, two case–control studies conducted in Cameroon found use of bed nets as a protective factor against Buruli ulcer (OR, 0.4; 95% CI, 0.2–0.9; *P* = 0.04 (ref. ^17^) and OR, 0.1; 95% CI, 0.03–0.3; *P* < 0.001 (ref. ^18^)).

However, case–control studies and entomological surveys alone are insufficient to implicate biological agents as vectors of pathogens. There are formal frameworks used in biomedicine, such as the Barnett criteria^3^, that build hierarchies of evidence to implicate a candidate disease vector. Here we build on this aforementioned research to formally address the Barnett criteria and to test the hypothesis that mosquitoes vector *M. ulcerans* to humans^14,19,20^. A summary of our findings is shown in Extended Data Table 1, including the new data shown in this study that specifically address criteria 1–3.

The research shown in this study is based on a substantial field survey of more than 65,000 mosquitoes undertaken over 4 months in 2019 and 2020, and four smaller ad hoc surveys conducted between 2016 and 2021. The study area was the Mornington Peninsula, an area of 350 km^2^, located 90 km south of Melbourne, the capital city of Victoria^9^ (Extended Data Fig. 1). The area was originally covered in low-lying coastal vegetation^9^, receives an average annual rainfall of 740 mm and sits at an average elevation of 60 m above sea level^21^. The Mornington Peninsula region continues to maintain among the highest incidences of Buruli ulcer in the world^22^, with a conservative local incidence estimate of 55 human cases per 100,000 population in 2022^4,23^. Here we used mosquito trapping, bacterial enrichment genome sequencing, mosquito blood-meal metabarcoding and spatial clustering analyses to build a hierarchy of evidence that supports mosquitoes as vectors of *M. ulcerans* from local wildlife reservoirs to humans.

## Results

### Mosquito surveys of the Mornington Peninsula

**Fig. 1:**
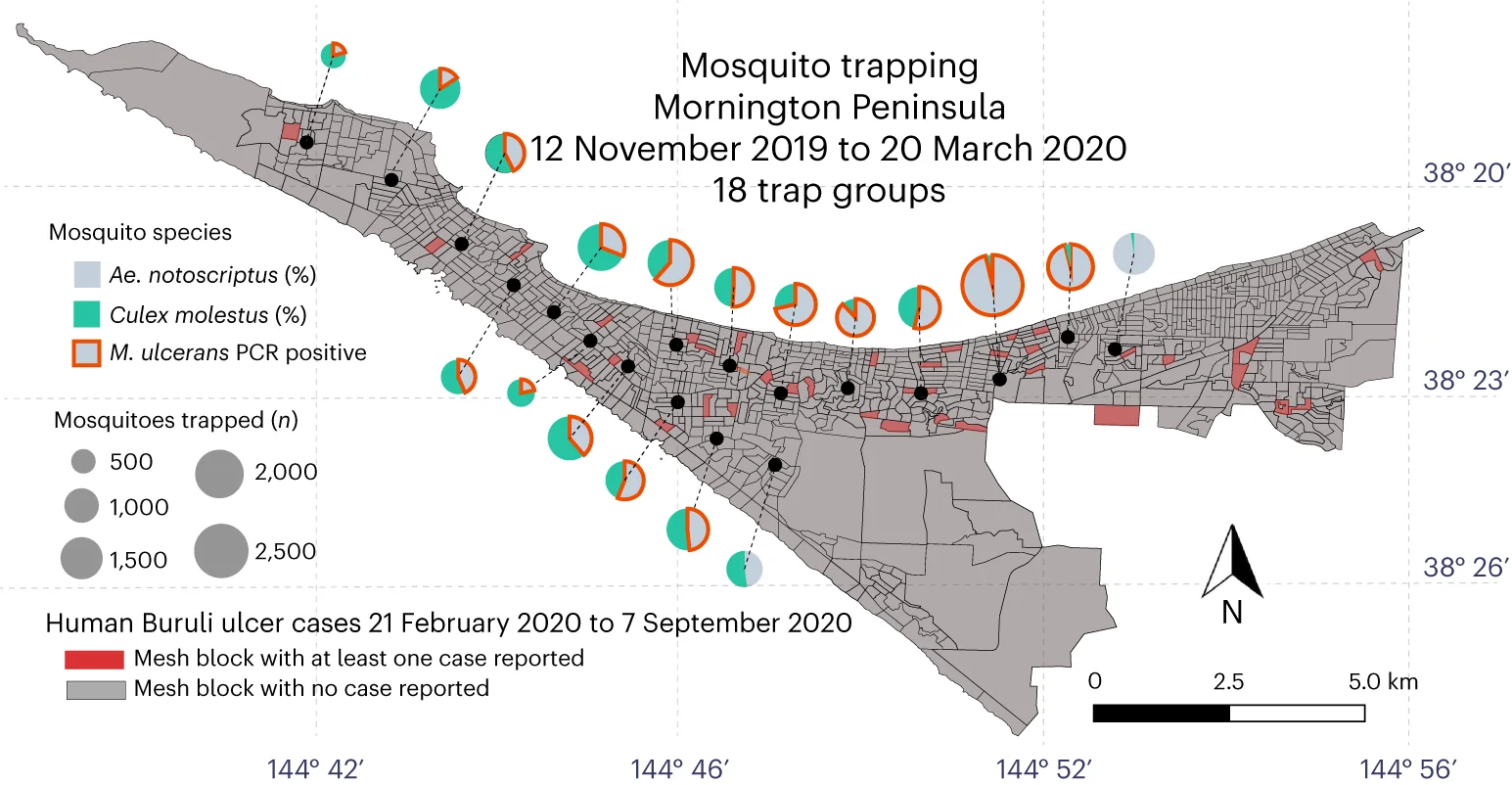
Dominant mosquito species distribution across the Mornington Peninsula. Map showing the proportional distribution of the two dominant mosquito species trapped during 2019 and 2020. The pie charts are an aggregation of the 180 different trap sites. Trap groups containing mosquitoes that were PCR positive for *M. ulcerans* are also indicated. The mesh-block statistical areas are also shown, with those in red containing at least one human Buruli ulcer case diagnosed in 2019–2020. Map boundaries from Australian Bureau of Statistics^69^ under a Creative Commons license CC BY 4.0.

A primary goal of this research was to test the hypothesis that mosquitoes are associated with *M. ulcerans* transmission, as reflected by IS*2404* PCR positivity at a certain frequency in areas of the Mornington Peninsula with human Buruli ulcer cases. A total of 73,580 mosquitoes were collected, consisting of 72,263 females (Table 1) and 1,317 males (Supplementary Table 1). The majority (90%) of these insects were collected in the large survey of 2019–2020 (Supplementary dataset 1). Across all 5 surveys, 26 different mosquito species were collected covering 6 genera (Table 1). The most dominant species identified during the 2019–2020 survey was *Culex molestus*, accounting for 42% of mosquitoes, followed by *Ae. notoscriptus* (35%) and *Culex australicus* (8%). Twenty-three other species comprised the remaining 15% of mosquitoes. The distribution of the two dominant species across the survey area is shown in Fig. 1. This mapping revealed an asymmetric distribution of each species, with *Ae. notoscriptus* dominant to the eastern end and *Culex molestus* dominant towards the western end of the peninsula (Fig. 1).

**Table 1:** Female mosquitoes trapped on the Mornington Peninsula and screened by IS*2404* PCR

Species	Number of mosquitoes/number of mono-species pools screened for *M. ulcerans* (number of pools positive for *M. ulcerans*, IS*2404*^a^)					
	December 2016 to April 2017	November 2017 to May 2018	December 2018 to May 2019	November 2019 to March 2020	February 2021 to March 2021	Total
*Aedes alboannulatus*	9/9	4/4	3/3	183/96	21/21	220/133
*Aedes australis*	0	0	0	1/0	0	1/0
*Aedes bancroftianus*	0	0	0	3/2	0	3/2
*Aedes camptorhynchus*	258/258	5/5	0	121/64 (1)	8/8	392/335 (1)
*Aedes clelandi*	0	0	0	41/32	0	41/32
*Aedes flavifrons*	1/1	0	0	31/26	13/13	45/40
*Aedes imperfectus*	0	0	0	266/226	0	226/226
*Ae. notoscriptus*	174/174 (2)	367/367 (8)	1,793/1,779 (15)	23,360/4,330 (14)	1,247/1,247 (7)	26,941/7,897 (46)
*Aedes rubrithorax*	9/9	27/27	230/230	1,402/335	45/45	1,713/646
*Aedes sagax*	0	0	0	2/2	0	2/2
*Aedes theobaldi*	0	0	0	1/1	0	1/1
*Aedes vigilax*	0	0	0	2/0	0	2/0
*Aedes vittiger*	0	0	0	2/0	0	2/0
*Anopheles annulipes*	1/1	24/24	4/4	751/376	1/1	781/406
*Anopheles atratipes*	0	0	0	2/0	0	2/0
*Coquillettidia linealis*	89/89	142/142	2/2	1,254/258	11/11	1,498/502
*Culiseta inconspicua*	0	0	0	2/2	3/3	5/5
*Culex annulirostris*	0	3/3	0	42/1	0	45/4
*Culex australicus*	3/3	198/198	17/17	4,910/534	33/33	5,161/785
*Culex cylindricus*	0	0	2/2	160/81	0	162/83
*Culex globocoxitus*	0	24/24	0	3,207/854	9/9	3,240/887
*Culex molestus*	61/61	29/29	384/377	28,107/3,308	1,465/1,465	30,046/5,240
*Culex orbostiensis*	0	0	0	131/69	0	131/69
*Culex quinquefasciatus*	3/3	257/257	37/37	1,098/401	163/163	1,558/861
*Tripteroides atripes*	0	0	0	18/0	12/12	30/12
*Tripteroides tasmaniensis*	0	0	0	15/13	0	15/13
Total	608/608 (2)	1,080/1,080 (8)	2,512/2,451 (15)	65,112/11,011 (15)	3,031/3,031 (7)	72,263/18,181 (47)

^a^Pool size ranged from 1 individual up to 15 individuals.

### *Ae. notoscriptus* not *Culex molestus* harbour *M. ulcerans*

The IS*2404* quantitative-PCR (qPCR) assay was used to infer the presence of *M. ulcerans* in association with mosquitoes^15^. Of the 73,580 mosquitoes trapped across all years, 18,610 (25%) were screened by IS*2404* qPCR for the presence of *M. ulcerans* (Table 1). *M. ulcerans* qPCR positives were observed in 53 pools or individuals of *Ae. notoscriptus*, with detections occurring in each year across all survey events (Fig. 1 and Table 1). Only one other mosquito species tested IS*2404* positive, which was a two-insect pool of *Aedes camptorhynchus* (Table 1). The positive association between *M. ulcerans* and *Ae. notoscriptus* compared with other mosquitoes, in particular the other abundant mosquito species, *Culex molestus*, was significant (*P* < 0.0001, Fisher’s exact test).

Subsets of *Ae. notoscriptus* were screened individually to better estimate the prevalence of *M. ulcerans*-positive mosquitoes in this species. Individually tested mosquitoes included all of the 2017–2018 collection comprising 367 of 367 screened individuals with 8 positives (2.2%), 448 of 4,330 screened individuals of the 2019–2020 collection with 3 positives (0.67%), and all 1,247 individuals of the 2021 collection with 7 positives (0.56%) (Supplementary Table 2). Thus, on the basis of screening individual insects, 18 of 2,062 (0.87%) *Ae. notoscriptus* were IS*2404* PCR positive. All other *Ae. notoscriptus* and other mosquito species in this study were screened in pools (up to 15 insects per pool) with 24 of 747 *Ae. notoscriptus* pools (3%) being PCR positive (Supplementary Table 3).

Of the 46 *Ae. notoscriptus* that tested positive for IS*2404*, 26 (56%) were confirmed positive for IS*2606* and 31 (67%) for KR, with 24 (52%) pools positive by all three qPCR assays (Supplementary Table 2). Of the 46 IS*2404*-positive *Ae. notoscriptus*, 8 (17%) pools were not tested using the IS*2606* assay and 1 (2%) pool was not tested using the KR assay due to limited template DNA available. The average cycle threshold (Ct) value for IS*2404*-positive *Ae. notoscriptus* was 36.00 (range, 29.74–39.65). The average Ct value for IS*2606* was 37.73 (range, 32.49–45.00) and the average Ct value for KR was 34.19 (range, 28.52–39.26). With reference to an IS*2404* qPCR standard curve (Supplementary Fig. 2), we estimated the *M. ulcerans* burden per mosquito. The mean bacterial genome equivalents (GE) per insect was 294 GE (range, 11–4,200; Supplementary Table 3). The MLE of estimated infection rate for all *Ae. notoscriptus* was 5.88 (95% CI, 4.37–7.76) based on the *M. ulcerans* IS*2404*-qPCR-positive mosquitoes per 1,000 tested for all *Ae. notoscriptus* tested over the years and 2.96 (95% CI, 0.17–14.19) for *Aedes camptorhynchus* (Table 2).

**Table 2:** *M. ulcerans*-positive pools tested by IS*2404* qPCR

Species	Number of mosquitoes	Number positive/number pools tested	MLE^a^	95% CI
*Aedes alboannulatus*	133	0/97	0	0–27.71
*Aedes bancroftianus*	2	0/2	0	0–657.62
*Aedes camptorhynchus*	335	1/85	2.96	0.17–14.19
*Aedes clelandi*	32	0/3	0	0–67.97
*Aedes flavifrons*	40	0/16	0	0–78.10
*Aedes imperfectus*	226	0/27	0	0–14.96
*Ae. notoscriptus*	7,897	46/2,937	5.88	4.37–7.76
*Aedes rubrithorax*	646	0/220	0	0–5.84
*Aedes sagax*	2	0/1	0	0–545.52
*Aedes theobaldi*	1	0/1	0	0–793.45
*Anopheles annulipes*	406	0/148	0	0–9.16
*Coquillettidia* *linealis*	502	0/139	0	0–7.43
*Culiseta inconspicua*	5	0/4	0	0–408.11
*Culex annulirostris*	4	0/3	0	0–450.75
*Culex australicus*	785	0/150	0	0–4.76
*Culex cylindricus*	83	0/38	0	0–41.60
*Culex globocoxitus*	887	0/150	0	0–4.22
*Culex molestus*	5,240	0/609	0	0–0.73
*Culex orbostiensis*	69	0/9	0	0–42.95
*Culex quinquefasciatus*	861	0/237	0	0–4.39
*Tripteroides atripes*	12	0/9	0	0–230.35
*Tripteroides tasmaniensis*	13	0/10	0	0–215.26

^a^MLE of *M. ulcerans* per 1,000 mosquitoes trapped in the Mornington Peninsula. MLE bias was corrected when one or more pool was positive.

### *M. ulcerans* genomes from mosquitoes, possums and people match

**Fig. 2:**
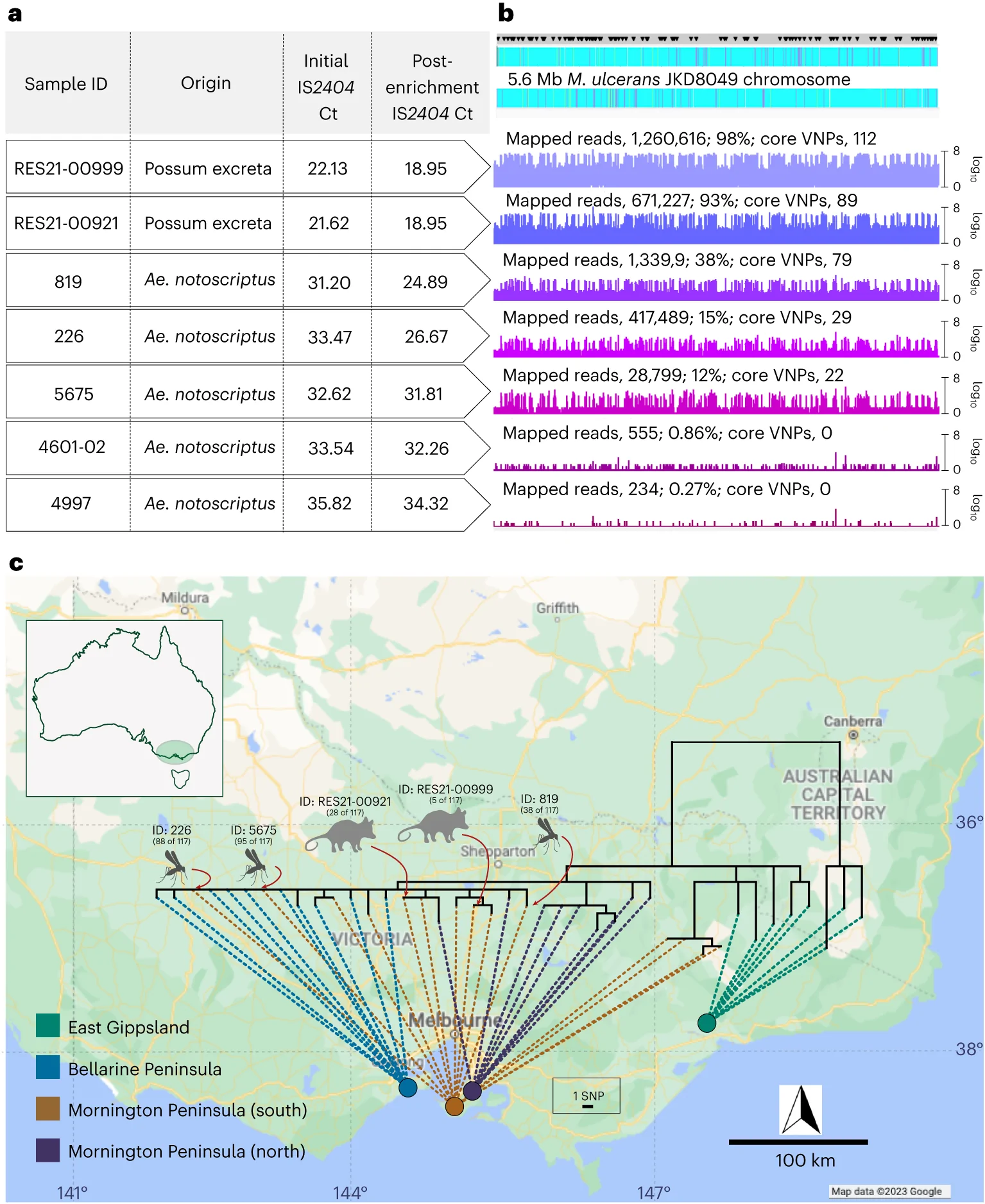
*M. ulcerans* genome from human Buruli ulcer cases compared with sequences recovered from possum excreta and mosquitoes on the Mornington Peninsula. **a**, Summary of IS*2404* qPCR screening of primary samples and the sequence-capture libraries pre- and post-enrichment. Note that possum excreta were enriched as barcoded sequence library pools so they share the same pre- and post-enrichment Ct values. **b**, Artemis coverage plots depicting sequence-capture reads mapped to the *M. ulcerans* JKD8409 chromosome from possum excreta samples and three qPCR-positive mosquitoes (labels are given as numbers of mapped reads; percentage of total chromosome bases mapped; number of core variable nucleotide positions (VNPs) covered). The grey horizontal bar above the chromosome map shows the sites of 117 core SNPs (black inverted triangles). **c**, Maximum likelihood phylogeny inferred from an alignment of 117 core genome SNPs using reads mapped to the JKD8049 reference chromosome, and with tips aligned with environmental sample origin or patient origin. The dataset includes reads from the 5 environmental samples listed in **a** with >21 VNPs, and a reference collection of 36 *M. ulcerans* genomes representing the genomic diversity of the *M. ulcerans* population in southeastern Australia^24^. The shortest vertical branch length represents a single SNP difference, as per the scale bar. Map boundaries in **c** from Google Maps under a Creative Commons license CC BY 4.0.

Genomic epidemiological studies have shown there are characteristic single-nucleotide-polymorphism (SNP) signatures associated with *M. ulcerans* clinical isolates from specific endemic areas in southeastern Australia^24^. To test if the *M. ulcerans* genotypes present in mosquitoes from our study area matched those found in possum excreta and human Buruli ulcer cases in the same region, we conducted genome sequence enrichment. As reported in other sequence enrichment studies, we observed decreasing genome sequence recovery with decreasing pathogen load, as indicated by increasing IS*2404* Ct values (Fig. 2a)^25^. Nevertheless, DNA sequence reads were obtained from five IS*2404*-positive mosquitoes and two IS*2404*-positive possum excreta specimens, the latter specimens collected as part of a large field survey of *M. ulcerans* in Australian native possum excreta in the region^26^. Although DNA sequence reads were obtained across the length of the 5.6 Mbp *M. ulcerans* reference chromosome, for all 5 mosquitoes and the 2 possum excreta specimens, only the excreta specimens (IS*2404* Ct values <23) and 3 of the 5 mosquito DNA extracts (identifier (ID) 5675, Ct 32.62; ID 226, Ct 33.47; ID 819, Ct 31.20) yielded sufficient *M. ulcerans* reads for SNP calling (Fig. 2b).

To assess the relatedness of *M. ulcerans* genomes from mosquito and excreta to human *M. ulcerans* isolates, we inferred a phylogeny using a 5.6 Mbp local *M. ulcerans* reference chromosome and sequence reads from 36 published clinical *M. ulcerans* genomes from southeastern Australia^24^, based on a core genome alignment with 117 SNP positions. These 36 genomes were selected because they spanned our previously reported population structure of *M. ulcerans* in this locale (Supplementary Data 2)^24^. Alignment of the five mosquito and two excreta sequence-capture datasets revealed reads mapping across the length of the *M. ulcerans* chromosome—confirming the presence of the pathogen on (or in) *Ae. notoscriptus* and in the possum faecal material (Fig. 2b).

**Extended Data Fig. 2:**
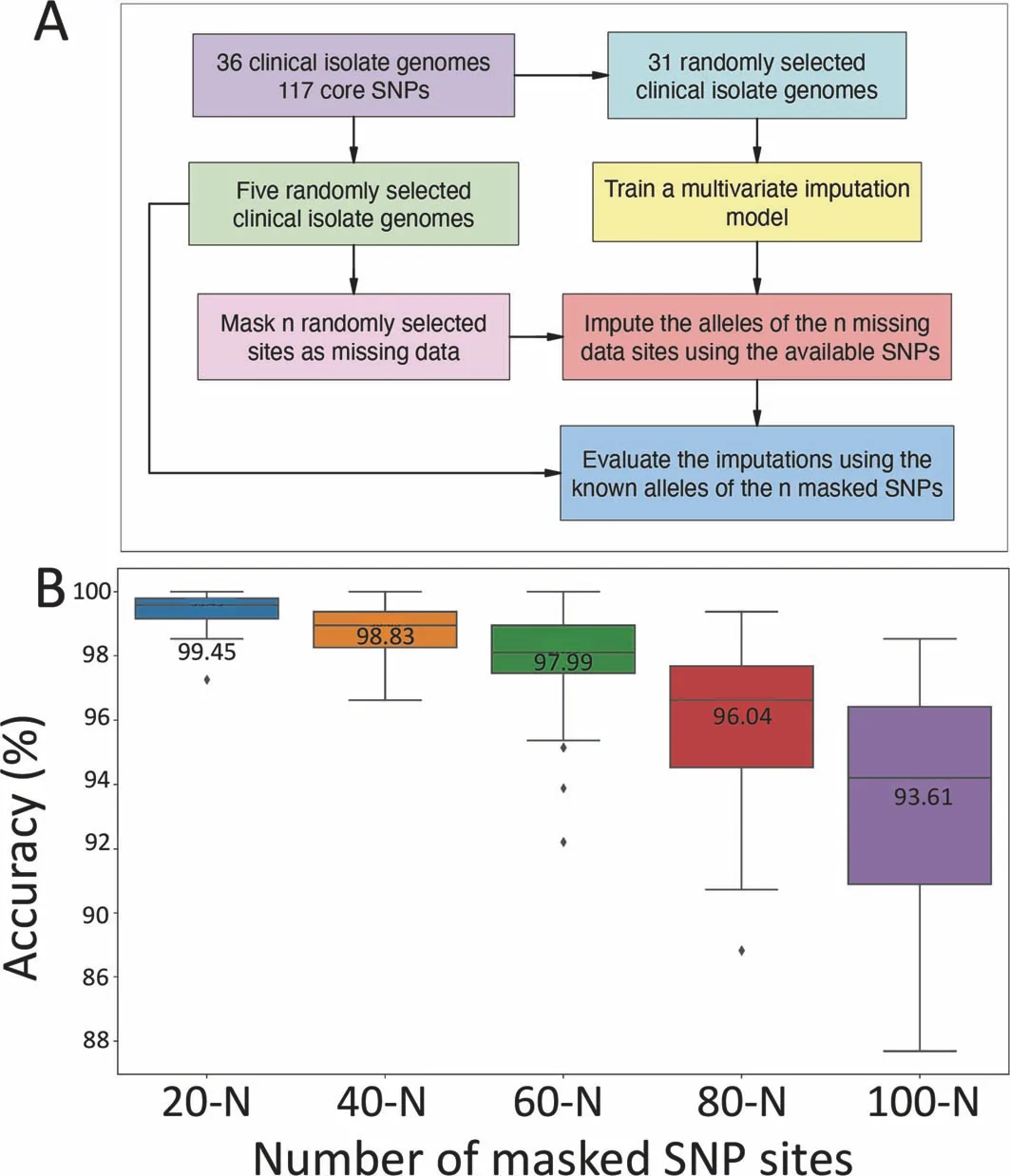
Validation of allele imputation approach to assess robustness to level of missing data and genome selection. (**a**) Flow diagram of imputation method. (**b**) Boxplots showing the distribution of imputation accuracy across 100 replicates for varying numbers of *M. ulcerans* chromosome SNP sites masked as missing data for five randomly selected *M. ulcerans* clinical isolate genomes. The line inside each box represents the median accuracy and the annotated numbers above each box indicate the mean accuracy in percentage. The whiskers extend to 1.5× the interquartile range. Source data

**Extended Data Fig. 3:**
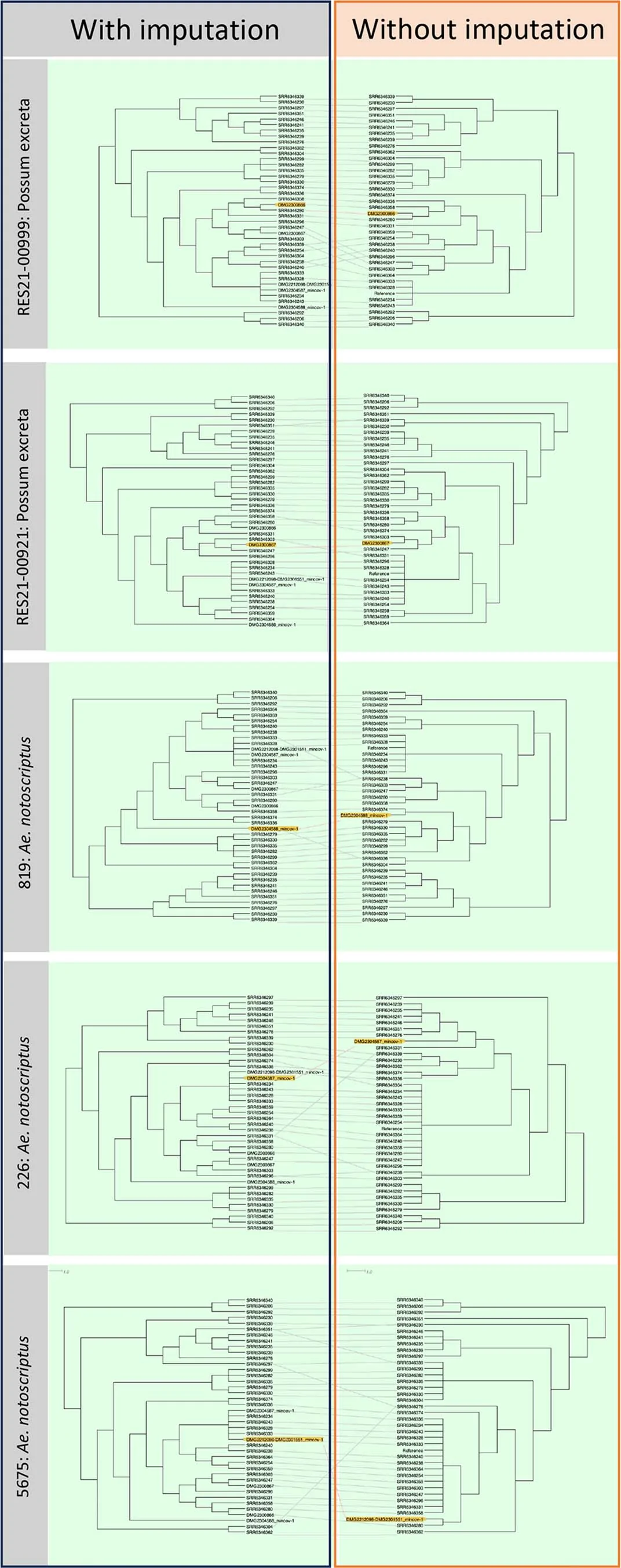
Tanglegrams analysis showing impact of imputation on phylogenetic inference. Depicted are tanglegrams (Dendroscope) showing phylogenies inferred with imputed SNP alignments containing each of the five, individual sequence enrichment and 36 clinical isolate *M. ulcerans* genome sequences compared to phylogenies of the individual sequence enrichment genomes and 36 clinical isolates without imputation. Yellow highlighted tips show the sequence enrichment genomes. Branch lengths transformed and presented as cladograms.

Despite chromosome-wide read mapping for the seven sequence-capture datasets, the relatively low and incomplete coverage meant that these datasets did not have reads spanning all 117 variable loci (range, 22–112 sites; Fig. 2b). Thus, to enable the inclusion of the sequence-capture datasets into a phylogenomic analysis of 117 core SNPs, a multivariate statistical approach using an imputation model was used (Extended Data Fig. 2). The model was trained on the 117 SNPs from the core genome alignment of all 36 clinical isolates to predict with high confidence (impute) the missing SNP alleles of the sequence-capture datasets (Methods). To validate the model, we initially masked 95 random SNP sites as missing data for a random set of 5 of the 36 clinical isolates, leaving 22 SNPs. Using the multivariate imputation model trained on the full 117 sites of the remaining 31 clinical isolate genomes, we predicted the missing alleles of the 5 masked clinical isolates (Extended Data Fig. 2). This approach yielded a mean accuracy of 97% in correctly predicting missing alleles, given the 22 available SNPs. To ensure the model was robust, we randomly selected five *M. ulcerans* clinical isolate genomes and then varied the number of masked chromosome SNP sites to simulate missing data and replicated this process 100 times. This analysis showed the high performance of the imputation model, with a mean accuracy of 94% when 100 of 117 SNP sites were randomly masked and then imputed (Extended Data Fig. 2). Using the validated imputation model to predict the missing alleles for the five sequence-capture datasets and create a sequence alignment (Supplementary Data 3), a maximum likelihood tree was inferred using all 117 variable sites with location of tree tips aligned with geographical origin. This phylogeny showed possum excreta and mosquito *M. ulcerans* genotypes co-clustered with each other and with human *M. ulcerans* isolates from the Mornington Peninsula, with 0–5 SNP differences between any pairwise comparison (Fig. 2c). This pattern is consistent with a shared transmission cycle between possums, mosquitoes and humans. Individual phylogenies inferred with and without SNP allele imputation showed that imputation did not create artefactual tree topologies (Extended Data Fig. 3).

### Genetic characterization of *Ae. notoscriptus*

To assess if *M. ulcerans* presence was associated with a particular clade of *Ae. notoscriptus* on the Mornington Peninsula, we compared cytochrome *c* oxidase subunit I (*COI*) gene sequences for 18 *M. ulcerans*-positive (confirmed by all IS*2404*, IS*2606* and KR qPCR assays) *Ae. notoscriptus* and 19 *M. ulcerans*-negative *Ae. notoscriptus*. On the basis of the *COI* phylogenetic tree, *Ae. notoscriptus* from the Mornington Peninsula spanned the three previously identified clades and there was no association between *M. ulcerans* and a particular mosquito lineage (Supplementary Fig. 1)^27^.

### IS*2404* PCR screen of arthropods other than mosquitoes

We also investigated the association of *M. ulcerans* with arthropods other than mosquitoes in the region using yellow sticky traps (YST) and sticky ovitraps (SO). A total of 21,000 specimens were collected and sorted from 278 YST and 33 SO. We were able to classify 2,696 specimens as insects that may bite or pierce human skin (Extended Data Table 2). Flies were the largest group collected on the sticky traps. YST collected more insects than the SO, but this was proportional to the number of traps set (Extended Data Table 2). Of the 2,696 insects screened by PCR, only 2 flies tested positive for *M. ulcerans* (Supplementary Table 2). Both flies had high Ct values for IS*2404* (Ct 35.92 and 37.54) and each sample was only confirmed by either the IS*2606* or the KR assay (Supplementary Table 2), but not with all three assays. Both insects were blow flies (*Calliphora hilli* Patton) based on morphology and sequencing of the *COI* region, with sequences having >99.86 nt identity to *Calliphora hilli*.

### Mosquito blood-meal analysis

**Fig. 3:**
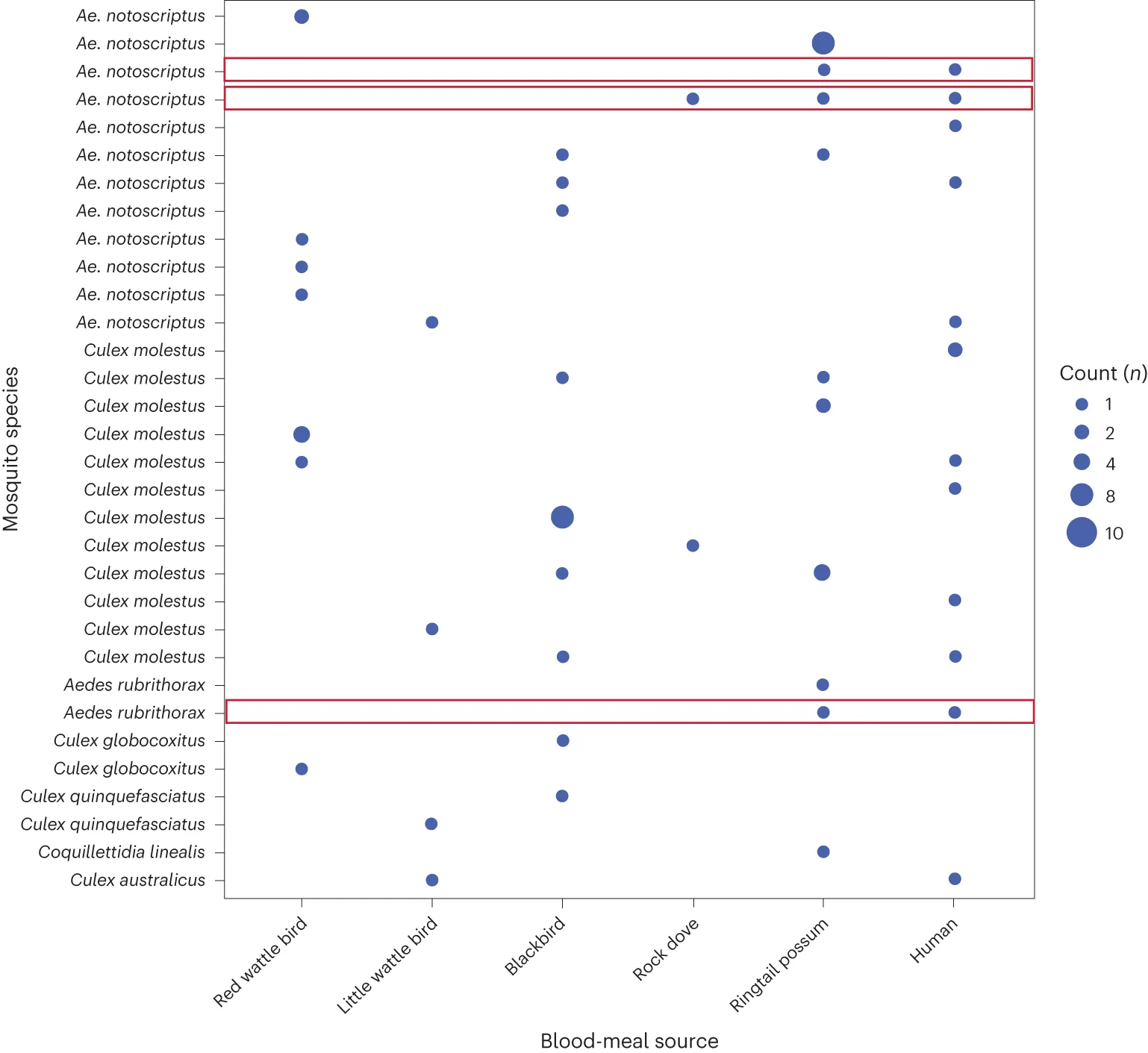
Mosquito blood-meal analysis. Summary of *cytb* gene sequences from the 36 blood-fed mosquitoes to identify host blood-meal sources. A blue circle indicates positive for host blood source; the larger the circle, the more individual mosquitoes with an identical blood-meal profile. Red boxes indicate individual mosquitoes that have dual blood meals for both humans and ringtail possum sources.

**Extended Data Fig. 4:**
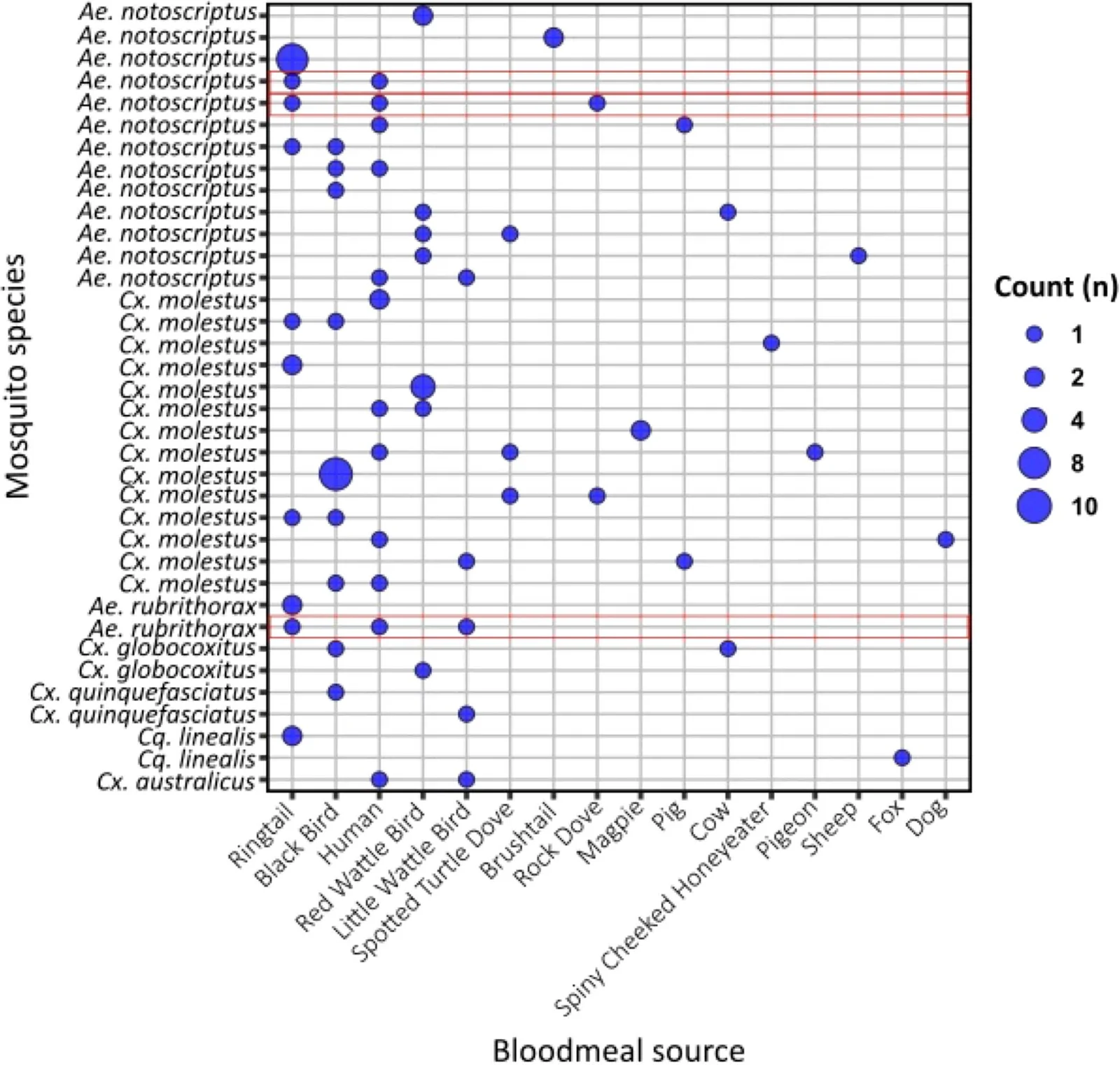
Mosquito bloodmeal analysis of all detected bloodmeal sources. A blue dot indicates positive for host blood source; the larger the dot, the more individual mosquitoes with an identical bloodmeal profile. Red boxes indicate individual mosquitoes which had dual bloodmeals from both humans and ringtail possum sources.

Of the mosquitoes collected, a proportion of individuals identified as being engorged (blood fed) were PCR screened and the resulting amplicon sequenced for cytochrome *b* (*cytb*) to identify host blood-meal sources. A total of 90 individual engorged mosquitoes were extracted, with 70 DNA preparations producing high-quality amplicons that were of sufficient concentration to permit Illumina amplicon sequencing. After quality filtering, 36 individuals were identified as having had a recent blood meal: 14 *Culex molestus*, 13 *Ae. notoscriptus*, 2 *Aedes rubrithorax*, 2 *Culex globocoxitus*, 2 *Culex quinquefasciatus*, 2 *Coquillettidia linealis* and 1 *Culex australicus*. Of the blood meals detected, the common ringtail possum was the most commonly identified with 20 detections across the 36 samples, followed by 17 blackbirds, 13 humans, 11 red wattle birds and 5 little wattle birds; a further 16 blood meals identified 10 minor host species (Fig. 3 and Extended Data Fig. 4). Dual blood meals were commonly identified, with 55% (20 of 36) of individuals having more than one blood meal identified. In addition, two mosquitoes had evidence of three different blood-meal sources within an individual insect. Three individuals (two *Ae. notoscriptus* and one *Aedes rubrithorax*) were also identified as having dual blood meals from ringtail possum and human origins (Fig. 3 and Extended Data Fig. 4).

### Clustering of mosquitoes, possums and human Buruli ulcer

**Fig. 4:**
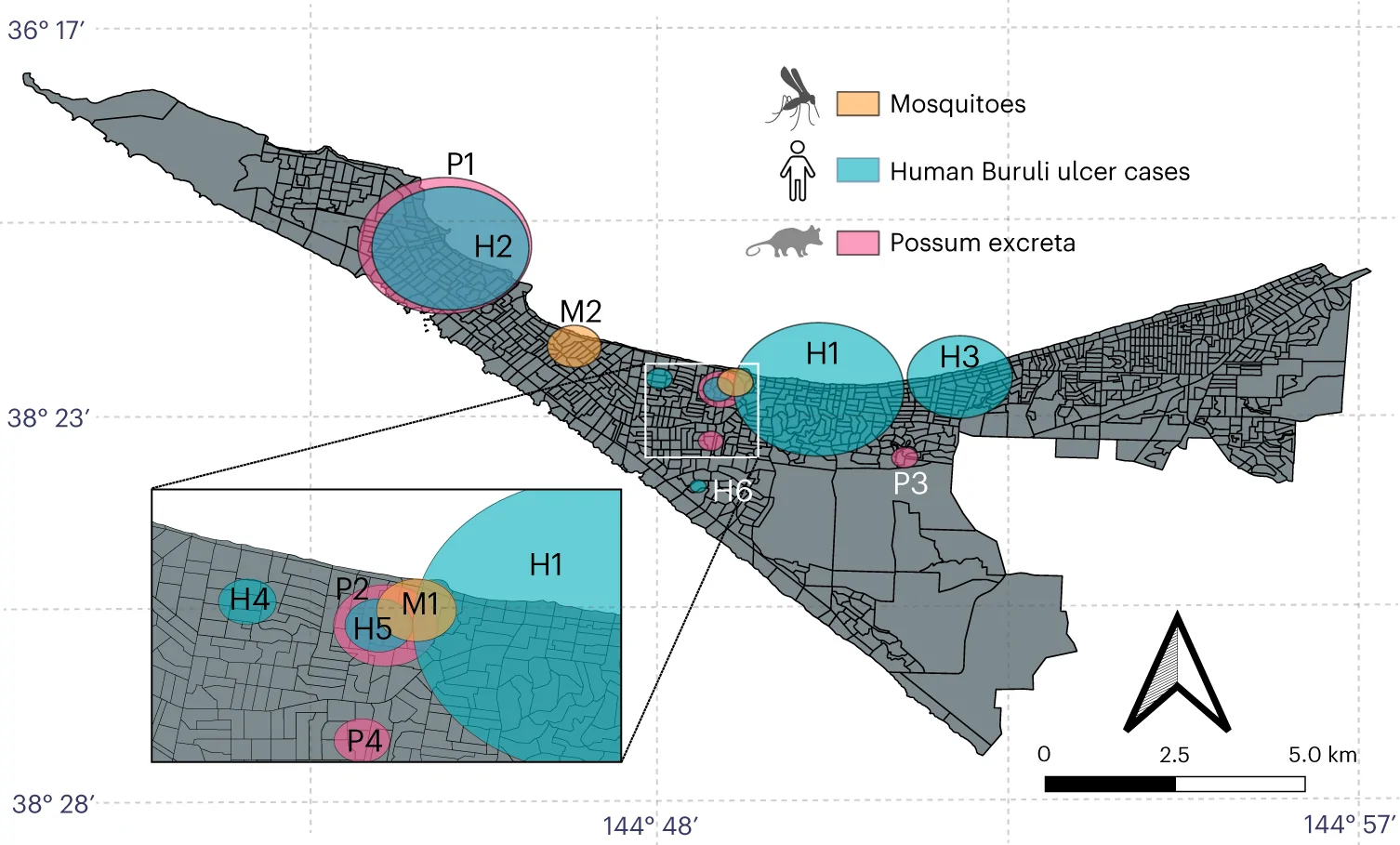
Spatial clustering of trapped mosquitoes, possum excreta and human Buruli ulcer cases on the Mornington Peninsula. Map showing the clusters identified by the three separate SaTScan analyses: (1) trapped mosquitoes (177 traps screened for IS*2404*; collected November 2019 to March 2020; denoted M1 and M2), (2) *M. ulcerans* detected in possum excreta collected during the summer of 2019 (December 2018 to February 2019; P1–P4) using data from a previous study, and (3) notified human Buruli ulcer cases from the study area in the years 2019–2020 (H1–H6). The inset shows an instance where all three analyses had overlapping clusters in the suburb of Rye. Map boundaries from Australian Bureau of Statistics^69^ under a Creative Commons license CC BY 4.0.

In our previous research, we showed a spatial association between possum excreta containing *M. ulcerans*, as detected by structured surveys, and clusters of human Buruli ulcer cases^26^. To expand on this finding, we investigated whether further spatial clustering associations could be detected by analysing qualitative qPCR (IS*2404*) data of trapped mosquitoes using SaTScan. Here three separate analyses were conducted: (1) human Buruli ulcer case data from individuals reported to have acquired the disease in the study area during 2019–2020, (2) qPCR data from possum excreta collected in a previous investigation (2018–2019) and (3) qPCR data from trapped mosquitoes (2019–2020). These analyses identified a single mosquito cluster, four possum clusters and six human Buruli ulcer clusters (Fig. 4 and Extended Data Table 3). Notably, one human Buruli ulcer cluster and two possum excreta clusters had higher numbers of observed Buruli ulcer cases or *M. ulcerans* detections, respectively, than expected if uniformly distributed (*P* < 0.05; Extended Data Table 3). Importantly, the analyses revealed an instance of triple-cluster overlap (mosquito, possum excreta and human) in the Mornington Peninsula suburb of Rye, where all three SaTScan analyses had an overlapping cluster (Fig. 4). A permutation test to assess the probability of these three categories (mosquitoes, possums and humans) overlapping showed that a triple-cluster overlap occurred with randomly rearranged location labels at a low frequency of 8.9%. This analysis adds support to a causal relationship between the presence of *M. ulcerans* in possums and mosquitoes and with humans contracting Buruli ulcer in the Mornington Peninsula suburb of Rye.

## Discussion

Using insect field surveys augmented with genomics, we have addressed Barnett criteria 1–3 (Extended Data Table 1) to add to a hierarchy of evidence implicating mosquitoes as the principal vectors in the spread of *M. ulcerans* from the environment to humans^3,14,16,19,20^. Our survey area was the Mornington Peninsula, where cases of human Buruli ulcer have increased progressively over the past 20 years^11^. Our key findings were that *M. ulcerans* was almost exclusively associated with one mosquito species in this region, *Ae. notoscriptus*, at a frequency of 0.87%. The estimated pathogen burden in each insect (Supplementary Table 2) was consistent with the reported low infectious dose for *M. ulcerans*^20^. Pathogen genomics provided support for a linked transmission chain between mosquitoes, native possums and humans. Metabarcoding mosquito blood-meal analysis revealed dual feeding by individual mosquitoes on both possums and humans, providing an example of a potential transmission pathway between infected possums and humans. Finally, spatial clustering analysis showed overlap between clusters of possums shedding *M. ulcerans*, mosquitoes harbouring *M. ulcerans*, and human Buruli ulcer cases, reinforcing the importance of possums and mosquitoes in the spread of *M. ulcerans* to humans.

A role for mosquitoes in the transmission of *M. ulcerans* was initially revealed in entomological surveys in the mid-2000s on the Bellarine Peninsula and then more recently in Far North Queensland^14,19,28^. Other observations that supported a role for mosquitoes in transmission included an analysis of the Buruli ulcer lesion location on the human body, which included more than 600 human Buruli ulcer cases, and showed a preponderance of lesions on ankles, elbows and backs of legs. These areas are frequently exposed and insects may preferentially blood-feed there^29^. Laboratory studies under one transmission scenario have also shown the competence of *Ae. notoscriptus* as mechanical vectors of *M. ulcerans* (Barnett criterion 4; Extended Data Table 1)^20^.

Of the ∼18,600 mosquitoes tested by IS*2404* PCR from 5 different survey periods in this study, *Ae. notoscriptus* (the second-most-abundant species trapped) was consistently positively associated with *M. ulcerans*. Where and how *M. ulcerans* is contaminating (or infecting) these mosquitoes remains to be determined. However, we made the somewhat unexpected observation that the most abundant mosquito species on the Mornington Peninsula, *Culex molestus*, was consistently IS*2404* PCR negative. This observation might be explained by a difference in the ecology of these two mosquito species, such as how (or where) they are encountering *M. ulcerans*. The apparent *Ae. notoscriptus*–*M. ulcerans* tropism might also indicate a specific biological interaction between this insect and the mycobacterium or, alternatively, a *Culex molestus* antagonism to mycobacterial carriage. Unfortunately, the ecology of mosquito subspecies in this region is generally poorly understood, including that of *Ae. notoscriptus*, which occurs as a series of genetically differentiated clades within Australia^27^.

To explore host sources for mosquitoes trapped in our study, we used metabarcoding sequencing of engorged mosquitoes^30^. Of the 90 blood-fed mosquitoes processed, 36 individual insects successfully had their host blood source identified. The 54 unidentified blood meals were probably due to degradation of the host DNA that occurs approximately 36 hours post-feeding^31^. Of the host blood sources identified, the common ringtail possum was the most commonly identified blood meal, followed by birds and humans, with dual blood meals observed in 55% of mosquitoes (Fig. 4 and Extended Data Fig. 3). Notably, three insects were determined to have blood meals from both common ringtail possums and humans. These insects consisted of two *Ae. notoscriptus* and one *Aedes rubrithorax*. The identification of these dual blood meals provides evidence that *Ae. notoscriptus* mosquitoes are blood-feeding between possums and humans within a relatively short timeframe. Dual blood meals may provide an opportunity for a mechanical transmission event of *M. ulcerans* from a possum with a Buruli ulcer teeming with *M. ulcerans*, from which a mosquito blood-feeds and then moves on to a nearby human. However, the observed positive association specifically between *Ae. notoscriptus* and *M. ulcerans* is not explained by our blood-meal analysis, which showed that several other mosquito species that were not *M. ulcerans* positive, such as *Culex molestus*, *Aedes rubrithorax* and *Coquillettidia linealis*, also fed on possums. An alternative source of *M. ulcerans* acquisition for *Ae. notoscriptus* might be from possum excreta contaminating the *Ae. notoscriptus* breeding sites, as this species breeds in small artificial containers^32^. If possum excreta, which can have a high concentration of *M. ulcerans*^10^, falls into these artificial breeding sites, then this may provide a means to contaminate the adult *Ae. notoscriptus* during eclosion and emergence on top of the water’s surface.

In common with other arthropod-borne bacterial pathogens, *M. ulcerans* also has the genomic hallmarks of niche adaptation^33,34^. For instance, the bacterial diseases vectored by blood-feeding arthropods—such as bubonic plague, spread to humans through the bite of fleas harbouring the bacterium *Yersinia pestis*^35^; the tick-borne infections such as Lyme disease, Rocky Mountain spotted fever and ehrlichiosis (among others) caused by *Borrelia burgdorferi*, *Rickettsia rickettsii* and *Ehrlichia* sp., respectively^36^; or tularaemia, caused by infection with *Francisella tularensis* spread through the bite of infected ticks (with some subspecies also spread by mosquitoes)^37–39^—are all caused by bacteria that have degenerating genomes. That is, as with *M. ulcerans*, their genomes bear the distinctive hallmarks of evolutionary bottlenecks and niche adaptation (plasmid acquisition, pseudogene accumulation and insertion sequence expansion), a genomic pattern thought indicative of a shared trajectory towards symbiosis with the arthropod host^40^. In addition, the absence of recombination and highly clonal population structure of *M. ulcerans* is aligned with the population structure of bacteria in ‘closed symbiosis’ with their host animal^41^.

Mosquitoes were not the only arthropods from which *M. ulcerans* were detected in the Mornington Peninsula. With over 20,500 arthropods collected using sticky traps (YST and SO), this study screened other insects that might broadly act as a mechanical vector, that is, an insect that is involved in the accidental transport of a pathogen^20,42^. *M. ulcerans* was detected on 2 of ∼1,800 flies tested, identified as blow flies (*Calliphora hilli*). Previous studies have screened limited numbers of other fly species, such as March flies (Tabanidae), with no positive detections^28^. *Calliphora hilli* is a native fly occurring along the Australian east coast and in South Australia^43^. It is a carrion breeder occurring year round throughout Victoria, with the female laying her eggs in decaying flesh from which the larvae emerge^43^. Possum carcasses are readily infested by *Calliphora hilli*^44^, which might explain how this fly species is becoming contaminated with *M. ulcerans*, as possums are wildlife reservoirs of *M. ulcerans*^9^. The likelihood of *Calliphora hilli* carrying *M. ulcerans* to humans is relatively low because this species rarely bites humans^45^.

In this study, we also used targeted enrichment genome capture to show beyond reasonable doubt the presence of *M. ulcerans* in association with mosquitoes^25^. Although the method lost sensitivity for IS*2404* qPCR Ct values >32, we generated sufficient genome coverage from three IS*2404*-PCR-positive mosquitoes and from two possum excreta specimens to show that the *M. ulcerans* genotypes of the captured genomes were identical to those associated with human Buruli ulcer cases in the study area, rather than Buruli ulcer cases linked to other regions (Fig. 2). We are now exploring the use of genome sequence enrichment to dissect the genomic epidemiology from the perspective of environmental sources, in particular to track the spread of Buruli ulcer in Australian native possums across the region^26^.

The triple-cluster overlap observed in our SaTScan analyses (Fig. 4) suggests that the spatial distribution of these three factors is likely to be closely linked and might be contributing to the persistence of Buruli ulcer in the Mornington Peninsula. Our findings highlight the importance of ongoing surveillance of possum and mosquito populations, which may provide critical insights into the epidemiology of Buruli ulcer in the region and inform targeted public health interventions to control the disease.

Our research has some limitations. Although we found a spatial association between the presence of *M. ulcerans* in possums and mosquitoes and with human Buruli ulcer cases, we cannot absolutely conclude causality or directionality. In addition, the very specific set of circumstances that have led to the rise of Buruli ulcer in temperate southeastern Australia restricts the generalizability of our results. One must be cautious to draw parallels with African Buruli ulcer endemic countries, for instance, where a highly susceptible mammalian reservoir equivalent to the Australian native possum is yet to be identified and evidence for mosquitoes as possible vectors is lacking^46^. Further investigations are needed to better understand the underlying mechanisms that drive the association between the presence of *M. ulcerans* in these different species and the development of human Buruli ulcer cases.

Despite these caveats, our collective research over more than 15 years makes it very clear that mosquitoes are probable vectors and native possums are major wildlife reservoirs of *M. ulcerans* in southeastern Australia. Mosquito surveillance with *M. ulcerans* screening coupled with mosquito control and public health messaging to avoid mosquito bites are practical interventions that would be expected to reduce the incidence of human Buruli ulcer.

## Methods

### Ethics

Ethical approval for the use in this study of de-identified human Buruli ulcer case location, aggregated at the mesh-block level, was obtained from the Victorian government Department of Health Human Ethics Committee under HREC/54166/DHHS-2019-179235(v3), ‘Spatial risk map of Buruli ulcer infection in Victoria’.

### Study site

Insects were collected from the Mornington Peninsula suburbs of Rye (population 8,416), Blairgowrie (population 2,313), Tootgarook (population 2,869) and Capel Sound (population 4,930)^47^ (Extended Data Fig. 1)^48^.

### Arthropod collection and identification

Mosquito-trapping campaigns used Biogent Sentinal (BGS) traps (Biogent) that were baited with dry-ice pellets to provide a source of CO_2_ over an intended 12 hour period. BGS traps were set out at dusk, and collected at dawn, in shaded locations on the grassed edges of the roadways in the study area. GPS locations for all traps were recorded, with data collection managed using Atlas of Medical Entomology (v.3.4.4; Gaia Resources). Trapped mosquitoes were knocked down with CO_2_ by placing the catch bag in dry ice before being transported back to the laboratory and kept at −20 °C until processing. Mosquito species were morphologically identified using a stereo dissecting microscope (SMZ800N; Nikon) and reference to taxonomic keys^49–51^. Data collection was managed using Microsoft Excel (v.16.73).

To assess the potential presence of *M. ulcerans* beyond mosquitoes, other arthropods were collected using YST and SO. Two YST and SO were placed in residential properties where householders had previously noted insect activity to the researchers^8^. The SO were placed on the ground and had hay grass infusion (3 Jack Rabbit (clover or lucerne) pellets (Laucke Mills) in 500 ml water) added to them, whereas the YST (Bugs for Bugs) were placed on the ground with a 14 cm plant tag plastic T-support (Garden City Plastics). Within 3–4 days of being set, residents were asked to pack up the YST and SO by covering the sticky card with a plastic film and return the sealed traps to the laboratory, where they were stored at −20 °C. Non-mosquito arthropods were morphologically identified to family level and, if PCR positive for *M. ulcerans*, were DNA barcoded for species confirmation by targeting the *COI* gene^52^.

### DNA extraction from mosquitoes

Mosquitoes were sorted by species per trap and by sex and then pooled in 2 ml O-ring tubes, with a maximum of 15 individuals in each pool. A subset of *Ae. notoscriptus* mosquitoes were also screened individually. Mosquitoes were homogenized with 10 mm × 1.0 mm zirconia/silica beads (BioSpec Products), with 597 µl of Buffer RLT and 2.8 µl of carrier RNA. Homogenization was performed using a TissueLyser II (Qiagen) at 30 oscillations per second for 100 s, repeated twice. Tubes were then centrifuged at 16,000*g* for 3 min. A 550 µl volume of supernatant was transferred into a 96-well deep-well plate, with extraction performed according to the protocol for the BioSprint 96 One-For-All Vet Kit (Qiagen). Every 11th of 12 wells in a 96-well plate was a blank DNA extraction control (7 in total) and a synthetic IS*2404*-positive control was spiked into 1 of these 7 wells to act as a positive extraction control. Extraction was performed on a KingFisher Flex Magnetic Particle Processor (Thermo Scientific).

### DNA extraction from arthropods

Arthropods other than mosquitoes collected on YST or SO were removed from the sticky cards and placed in 1.5 ml microtubes (Eppendorf). Insects were separated by family and trap location, and were pooled with a maximum of 10 individuals from each family per 1.5 ml tube. Samples were extracted non-destructively to allow species confirmation if positive detections occurred. DNA was extracted using the ISOLATE II Genomic DNA Kit (Bioline). Briefly, 25 µl of Proteinase K and 180 µl of Lysis Buffer GL was added to each tube with samples incubated overnight at 56 °C. Following incubation, the insects were removed and stored to allow for further morphological identification if required, with the DNA extraction completed on the incubation solution as per manufacturer instructions.

### Synthetic PCR positive control

A synthetic PCR positive control DNA molecule was designed to discriminate false positives due to contamination with positive control DNA versus the authentic IS*2404* amplicon. The synthetic positive control was designed to have an amplicon size of 120 bp to easily differentiate it from a true IS*2404* PCR positive (59 bp)^15^. The synthetic positive was added at the DNA extraction stage on all 96-well plates as a positive control for this step and for the subsequent qPCR. The additional DNA sequence used to construct the synthetic positive control was randomly selected from a DNA sequence unlikely to be in the laboratory, in this case, Irrawaddy dolphin (MK032252).

The synthetic positive control had the sequence 5′-TCCTAAAGCACCACGCAGCATCTATCGCGAGCTTAATCACCATGCCGCGTCCAACGCGATCCCCGCTCGGCAGGGATCCCTCTTCTCGCACCGGGCCACAATCCACTGGGGTCGCTATGA-3′ and was synthesized as a single-stranded DNA oligonucleotide (Sigma-Aldrich). The underlined regions indicate the forward primer, probe and reverse primer sequences, respectively. The synthesized IS*2404* synthetic positive was resuspended in nuclease-free water and diluted to 0.001 pM, with 2 µl being used for extraction and positive controls. To confirm the presence of a true positive as opposed to contamination, 5 µl of the qPCR product was added to 1 µl of DNA Gel Loading Dye 6X (Thermo Scientific) and run on a 2% agarose gel (TopVision Agarose Tablets; Thermo Scientific), with 1% SYBR Safe DNA Gel Stain (Invitrogen). The size of any positive IS*2404* detection was assessed against 2 µl of 100 bp DNA Ladder (Promega) and run at 50 V for 1.5 h before being visualized with an EZ Gel Documentation System (Bio-Rad). Before the screening of insects began, the synthetic positive control for IS*2404* was successfully designed and tested. By running the amplified PCR products on an agarose gel with the synthetic positive control and a real positive control, visual differentiation could be determined between a synthetic positive occurring at 120 bp and a true positive at 59 bp (Supplementary Fig. 2).

### Screening insects by qPCR for *M. ulcerans*

The qPCR screening was performed using three independent assays IS*2404*, IS*2606* and KR^15^. All samples were first screened with the IS*2404* qPCR; if a positive was detected, additional confirmation was attempted with IS*2606* and KR qPCR assays. Reactions were performed using 7.5 µl TaqMan Fast Universal PCR Master Mix (2X), no AmpErase UNG (Applied Biosystems), 1 µl of the primer–probe mix, 2 µl DNA and 4.5 µl nuclease-free water. A final primer–probe concentration for the IS*2404* assay was as follows: 250:650:450 nM for the forward primer, reverse primer, and probe; and 800:800:220 nM for the forward primer, reverse primer, and probe for the IS*2606* and KR assay. A 2 µl volume of the synthetic positive control was added for the IS*2404* reactions, whereas 2 µl of *M. ulcerans* DNA was used for IS*2606* and KR. All reactions included 6 no-template extraction controls and were run in a 96-well-plate format. Cycling conditions were as follows: denaturation at 95 °C for 2 min, followed by 45 cycles at 95 °C for 10 s and 60 °C for 30 s, with qPCR performed on a QuantStudio 5 Real-Time PCR System (Applied Biosystems). Positives were classified as reactions that produced a cycle quantification value less than 40. Data were analysed using the QuantStudio Design and Analysis Software v.1.4.3 with the ΔRn threshold set at 0.04 for IS*2404* and IS*2606*, and a ΔRn threshold of 0.1 for KR. The MLE per 1,000 mosquitoes tested (bias-corrected MLE for point estimation of infection rate and a skew-corrected score CI) was calculated from the pooled samples^53^. Fisher’s exact test for assessing the significance of differences in IS*2404* PCR positivity between mosquito species was calculated in R 4.0.2 (ref. ^54^).

All qPCR screening was performed blind with mixed-species 96-well plates. The synthetic positive control was added at the DNA extraction phase to one well of each plate and to qPCR plates to check that both extraction and qPCR detections were successful. All no-template controls (extraction and qPCR stage) were checked to ensure they remained negative, and that synthetic positive controls were detected for both the DNA extraction and qPCR stage in each run. Positive samples were run on agarose gels to confirm they were true positives and not contamination from the synthetic positive control.

An IS*2404* qPCR standard curve was prepared using 10-fold serial dilutions of *M. ulcerans* genomic DNA, with quadruplicate testing of each dilution. The DNA was extracted from *M. ulcerans* JKD8049 and quantified using fluorimetry (Qubit dsDNA HS; Thermo Fisher Scientific)^20^. A limit of detection was defined as the lowest dilution that returned a positive signal for all four replicates. GE were calculated based on the estimated mass of the *M. ulcerans* genome of 5.7 fg (ref. ^20^). IS*2404* Ct values were converted to GE to estimate bacterial load within a sample by reference to a standard curve (*r*^2^ = 0.9956, *y* = [−3.829ln(*x*) + 37.17]*Z*, where *r2* = correlation coefficient *y* = Ct and *x* = amount of DNA (in fg) and *Z* = the dilution factor; Supplementary Fig. 2). An IS*2404* qPCR standard curve was fitted using nonlinear regression in GraphPad Prism (v.9.5.1) (Supplementary Fig. 3).

#### *M. ulcerans* genome sequencing

Whole-genome sequencing was performed directly on DNA extracted from selected PCR-positive mosquito samples and possum excreta specimens using a hybridization capture approach, based on 120 nt RNA baits spanning the 5.8 Mbp chromosome of the *M. ulcerans* JKD8049 reference genome (BioProject ID PRJNA771185) (SureSelect Target Enrichment System; Agilent; Supplementary dataset 4) and the Illumina *Nextera Flex for Enrichment with RNA Probes* protocol^55^. Resulting sequence reads were submitted to National Centre for Biotechnology Information (NCBI) GenBank and are available under BioProject PRJNA943595 (Supplementary Table 2).

#### *M. ulcerans* SNP calling, SNP imputation and phylogenetic analysis

To compare genomic variations between *M. ulcerans* clinical isolate genome sequences and sequence-capture enrichment datasets, we mapped the sequence reads and called nucleotide variations using Snippy (v.4.4.5) against a finished *M. ulcerans* reference chromosome, reconstructed from a Victorian clinical isolate (JKD8049; GenBank accession NZ_CP085200.1; https://github.com/tseemann/snippy). Although standard parameters, including a minimum coverage of 10×, were used for the clinical isolates and two possum sequence-capture datasets, the mosquito sequence-capture datasets had lower read coverage, necessitating the adjustment of parameters. Thus, the minimum coverage threshold was lowered to 1× to facilitate SNP calling for the mosquito sequence-capture datasets. The resulting SNPs were combined with 117 SNPs obtained from a reference set of 36 *M. ulcerans* genomes that represented the previously defined population structure of the pathogen in Victoria^24^. Due to the low read coverage however, the number of core variable nucleotide positions mapped among the 5 sequence-capture datasets was variable (range, 22–112 variable nucleotide positions). To enable inclusion of the sequence-capture datasets that had missing SNP sites, we used a multivariate imputation approach, using the IterativeImputer function from scikit-learn^56^. The combined alignment of 117 core genome SNPs from the sequence-capture datasets and clinical isolate genomes served as the foundation for inferring a maximum likelihood phylogeny. This phylogeny was established using the GTR model of nucleotide substitution and executed with FastTree (v.2.1.10)^57^. The incorporation of R packages phytools (v.1.0-1)^58^ and mapdata (v.2.3.1)^59^ allowed for the alignment of tree tips against a base map, facilitating the visualization of geographical origins of the samples. Further details, including the code used for missing SNP imputation and phylogeographical analysis, can be found in our GitHub repository^60^.

### *Ae. notoscriptus* typing and species confirmation sequencing

Mosquito genotyping was performed by sequence comparisons of a partial fragment of the *COI* gene^52^. DNA was extracted using the above protocols. PCR was performed using 5 µl of 5× MyFi Reaction Buffer, 1 µl MyFi DNA polymerase, 5 µl DNA and primer concentrations^27^, with the reaction made up to 25 µl with nuclease-free water. Reaction conditions were as follows for *COI*: initial denaturation at 95 °C for 1 min, followed by 35 cycles at 95 °C for 20 s, 46 °C for 20 s and 72 °C for 60 s, before a final extension at 72 °C for 5 min. A 5 µl volume of the amplified PCR product was added to 1 µl of DNA Gel Loading Dye 6X (Thermo Scientific) and run on a 1% agarose gel (TopVision Agarose Tablets; Thermo Scientific), with 1% SYBR Safe DNA Gel Stain (Invitrogen). A 2 µl volume of 100 bp DNA Ladder (Promega) was added to confirm amplicon size and run at 100 V for 45 min. PCR products that produced bands of the correct size were purified using the ISOLATE II PCR and Gel Kit (Bioline), as per the manufacturer’s protocol, and submitted for sequencing using an Applied Biosystems 3730xl capillary analyser (Macrogen), with sequencing occurring on both strands. Sequences were analysed in Geneious Prime (v.2019.2.1) and trimmed to high-quality bases, aligned using ClustalW v.2.1 and trimmed to a consensus region, *Ae. notoscriptus* *COI* (874 bp) and for species identification *COI* (816–882 bp). Sequences were analysed using blastn against the NCBI database. *COI* sequences generated for species identification are available under accession numbers OQ600123–OQ6001234, and *COI* sequences for *Ae. notoscriptus* phylogenetics are under accession numbers OQ588831–OQ588867 (Supplementary Fig. 1).

### Mosquito blood-meal analysis

Ninety blood-fed mosquitoes were identified as having an engorged abdomen and still having a red pigment (Sella score 2–3), indicating a fresh blood meal, and were dissected with a sterile scalpel blade. Blood from the dissected abdomen was absorbed onto a 3 mm × 20 mm piece of a Whatman FTA card (Merck) and placed in a 2 ml tube. DNA was extracted from the FTA card using an ISOLATE II Genomic DNA Kit (Bioline) with a pre-lysis in 180 µl of Lysis Buffer GL and 25 µl Proteinase K for 2 hours before completing the extraction as per the manufacturer’s protocol. Extracted DNA was amplified for cyt*b* using primers previously described^61^, with MyTaq HS Red Mix (Bioline), and the thermocycling conditions used were as follows: 95 °C for 1 min, 30 cycles of 95 °C for 15 s, 50 °C for 20 s, 72 °C for 20 s and a final extension at 72 °C for 2 min. Negative extraction and negative PCR controls were included with each PCR reaction. A volume of 5 µl of the amplified products was run on a 1% agarose gel containing 0.1% SYBR Safe DNA gel stain (Invitrogen) and visualized with a G:BOX Syngene blue-light visualization instrument. If a band was visualized at ∼480 bp, the PCR product was purified using an ISOLATE II PCR and Gel Kit (Bioline).

PCR products were quantified using a Qubit (Invitrogen) fluorometer with an HS dsDNA kit. Sequencing libraries were prepared using 10 ng of purified PCR product with a NEXTFLEX Rapid XP DNA-Seq Kit (PerkinElmer) barcoded using NEXTFLEX UDI Barcodes (PerkinElmer). As a result, 70 blood-meal libraries were sequenced, along with 6 PCR negative controls and 3 extraction negatives, on a NovaSeq 6000 (Illumina), with 2 Gb requested per sample.

Sequence data were analysed by identifying poor-quality reads using Rcorrector^62^ and removed with TrimGalore v.0.6.5. De novo assembly was performed on the remaining sequence reads using Trinity v.2.8.6^63^. The resulting contigs were filtered to be between 400 bp and 480 bp and analysed using BLASTN against the nucleotide database publicly available on the NCBI website. The resulting hits were filtered to exclude those sequences that had <97% sequence identity to the database. Contigs identified as a host blood meal were confirmed by mapping raw reads using BWA-MEM v.0.7.17^64^. Sequence reads were submitted to GenBank (BioProject ID PRJNA943595).

### Mosquito phylogenetic analysis

Mosquito genotyping was performed with *COI* because this genetic marker has previously been used to identify potential cryptic species within *Ae. notoscriptus* and can provide better resolution than other markers such as *ND5*, *CAD* or *EPIC* (exon-primed intron crossing)^27^. Phylogenetic analysis was performed on trimmed consensus regions of *Ae. notoscriptus* *COI*. The substitution model was selected using jModelTest2 v.2.1.10, with the topology taking the best of nearest-neighbour interchange, subtree pruning and regrafting^65^. The most appropriate substitution model was selected based on the Akaike information criterion. Maximum likelihood trees were constructed in PhyML v.3.3.2 with 1,000 bootstrap replicates; the gamma distribution parameter was used to estimate rate variation across sites^66^. The Hasegawa–Kishino–Yano (HKY) substitution model was selected for the *COI* tree.

### Geographical data acquisition and spatial cluster analysis

The population map was created using QGIS geographical information system software (v.3.16.7)^67^, using a 1 km^2^ population grid^68^ with 2011 Victorian mesh-block data. Since 2004, Buruli ulcer has been a notifiable condition in Victoria, requiring health department reporting by doctors and laboratories. De-identified case notification data of patients with Buruli ulcer who had laboratory-confirmed *M. ulcerans* infection and who lived on the Mornington Peninsula during the years 2019–2020 were provided by the Victorian Department of Health. The cases were defined as patients with a clinical lesion that was diagnosed using IS*2404* qPCR and culture^15^. To conduct high-resolution spatial analyses, the data were aggregated at the mesh-block level, the smallest geographical census units which typically contain 30–60 dwellings. The 2011 Victorian mesh-block boundaries and the Victorian mesh-block census population counts datasets were obtained from the Australian Bureau of Statistics website^69^. The datasets were joined using the unique mesh-block IDs using QGIS (v.3.16.7)^67^. The latitude and longitude (projected in GDA94) were derived from the centroids of the mesh-block polygon. The dataset was then downsampled to include only the Mornington Peninsula study area, specifically the Point Nepean and Rosebud–McCrae Australian Bureau of Statistics level 2 Statistical Area.

SaTScan v.10.1.0 (ref. ^70^) was used to identify spatial clusters among trapped mosquitoes positive for *M. ulcerans*, possum excreta positive for *M. ulcerans* and human Buruli ulcer cases. The software searches for instances where the observed number of spatial incidences exceeds the expected number within a circular window of varying size across a defined study area. A log-likelihood ratio statistic is calculated for each window by comparing the number of observed and expected cases inside and outside the circle against the assumption of randomly distributed cases. In addition to the most likely cluster, there are usually secondary clusters with almost as high likelihood that substantially overlap with the primary clusters. These secondary clusters can be indicative of subclusters within the primary cluster or potentially distinct clusters that are spatially adjacent to the primary cluster. The Mornington Peninsula surveillance data used in these analyses consisted of trapped mosquitoes (177 traps screened for IS*2404* collected 12 November 2019 to 20 March 2020), *M. ulcerans* detected in possum excreta collected during the summer (December to February) of 2019 using data from a previous study^26^ and notified human Buruli ulcer cases from the study area in the years 2019–2020. The use of possum excreta collected during the summer was appropriate as Buruli ulcer transmission is most likely to occur during that time of year^71,72^. For each of the three data sources (trapped mosquitoes, possum excreta and human cases), the null hypothesis assumes that *M. ulcerans* detections or Buruli ulcer cases are uniformly distributed across the study area, where the alternative hypothesis suggests that there may be certain locations with higher rates than expected if the risk was evenly distributed. Primary and secondary clusters were accepted only if the secondary clusters did not overlap with previously reported clusters with a higher likelihood. Given that the trapped mosquito and possum excreta IS*2404* PCR results were binary (positive or negative), a Bernoulli model was used to scan for spatial clusters, with the maximum cluster size set to 50% of the population size. The human Buruli ulcer case data aggregated at the mesh-block level varied in number, with some mesh blocks having zero cases and others having one or more. We applied the Poisson probability model to the notified Buruli ulcer case counts, using a background population at risk that was derived from the 2011 population census. The maximum cluster size was limited to 14,481 individuals, 10% of the total population at risk. To determine the likelihood of a triple-cluster overlap between the three SaTScan analyses occurring by chance, we conducted a permutation test^73^. In each of 10,000 iterations, the geographical coordinates for each variable were randomly shuffled. The number of SaTScan clusters with triple overlap was determined using the sf package^74^ in the R statistical programming language^54^.

### Reporting summary

Further information on research design is available in the Nature Portfolio Reporting Summary linked to this article.

## Supplementary information

## Source data

## Figures and Tables

**Extended Data Table 1:**
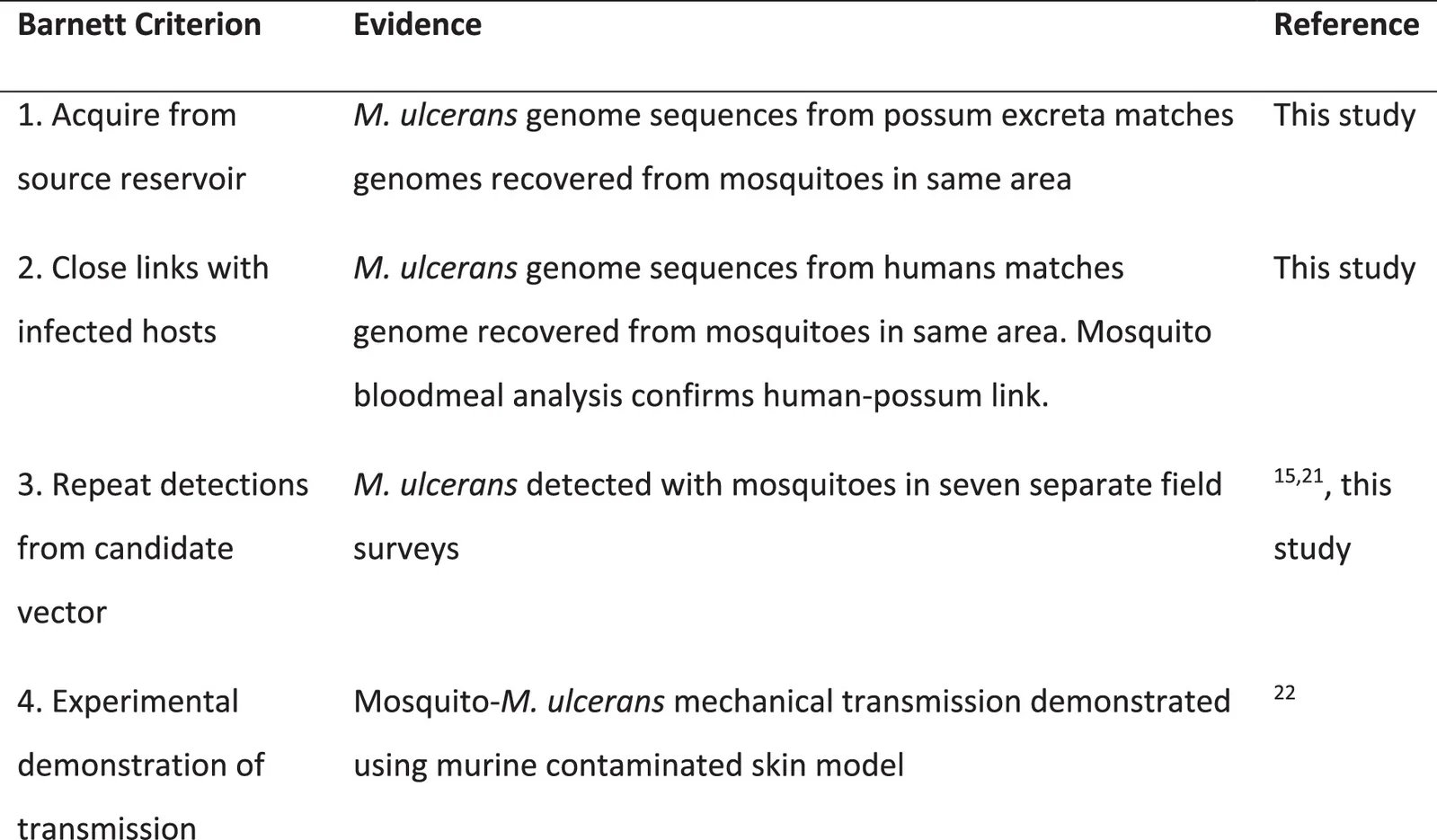
Summary of evidence to indict mosquitoes as vectors of *M. ulcerans*

**Extended Data Table 2:**
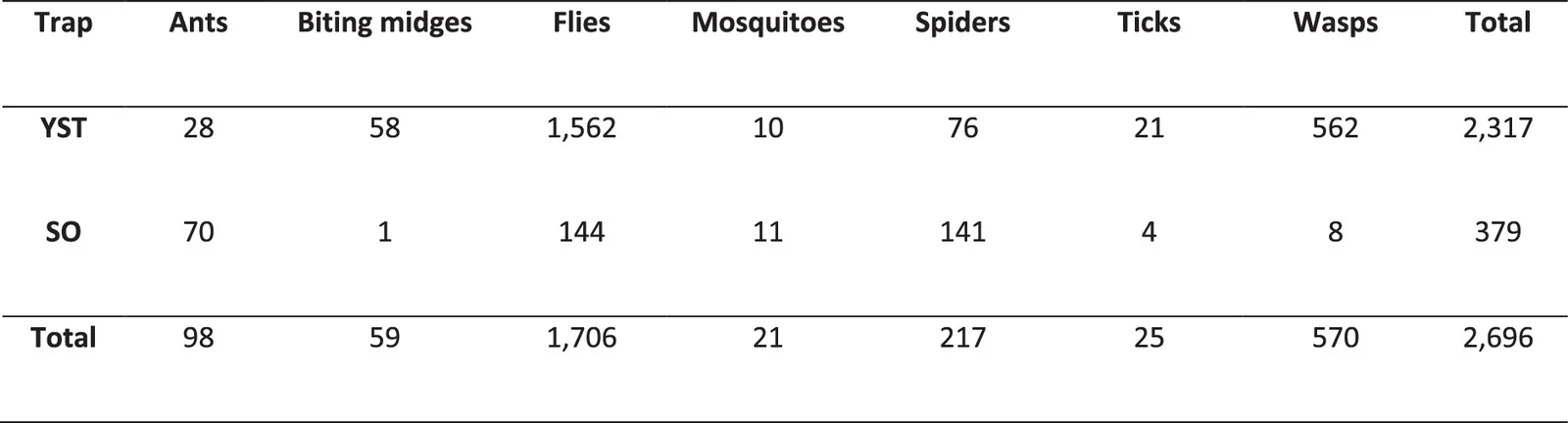
Number of arthropods collected on Yellow Sticky Traps (YST) and Sticky Ovitraps (SO) on the Mornington Peninsula that might bite or pierce human skin

**Extended Data Table 3:**
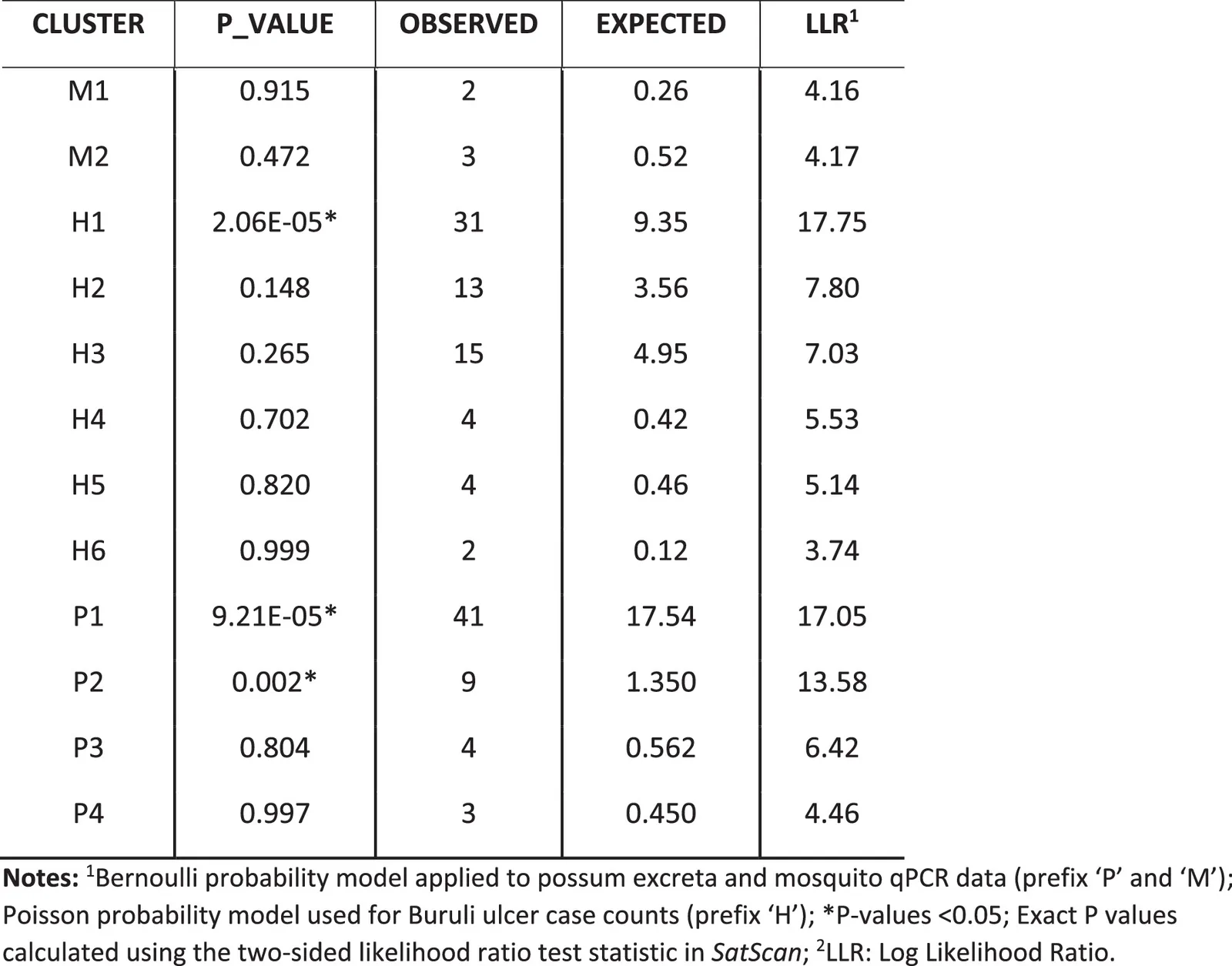
Summary of the SaTScan cluster analysis

## Data Availability

DNA sequencing data generated in this project are available under the following NCBI GenBank accession numbers: PRJNA943595, PRJNA943595, NZ_CP085200.1, OQ600123, OQ6001234, OQ588831–OQ58883167. Source data are provided with this paper.

## Code availability

The code is available via Github at https://github.com/abuultjens/BU-3-way-SatScan for SatScan analysis and https://github.com/abuultjens/Mosquito_possum_human_genomic_analysis.for sequence enrichment data analysis.

## References

[1] MacCullum, P., Tolhurst, J. C., Buckle, G. & Sissons, H. A. A new mycobacterial infection in man. *J. Pathol. Bacteriol.* **60**, 93–102 (1948). 18935206

[2] World Health Organization. Buruli ulcer (*Mycobacterium ulcerans* infection) https://www.who.int/news-room/fact-sheets/detail/buruli-ulcer-(mycobacterium-ulcerans-infection) (2022).

[3] Merritt, R. W. et al. Ecology and transmission of Buruli ulcer disease: a systematic review. *PLoS Negl. Trop. Dis.* **4**, e911–e911 (2010). 21179505 10.1371/journal.pntd.0000911 PMC3001905

[4] Department of Health and Human Services (Victoria). Interactive infectious disease surveillance reports https://www2.health.vic.gov.au/public-health/infectious-diseases/infectious-diseases-surveillance/interactive-infectious-disease-reports (2021).

[5] Janssens, P. G., Quertinmont, M. J., Sieniawski, J. & Gatti, F. Necrotic tropical ulcers and mycobacterial causative agents. *Trop. Geogr. Med* **11**, 293–312 (1959). 14406764

[6] Clancey, J. K., Dodge, O. G., Lunn, H. F. & Oduori, M. L. Mycobacterial skin ulcers in Uganda. *Lancet* **2**, 951–954 (1961). 13879648 10.1016/s0140-6736(61)90793-0

[7] Muleta, A. J., Lappan, R., Stinear, T. P. & Greening, C. Understanding the transmission of *Mycobacterium ulcerans*: a step towards controlling Buruli ulcer. *PLoS Negl. Trop. Dis.* **15**, e0009678 (2021). 34437549 10.1371/journal.pntd.0009678 PMC8389476

[8] Blasdell, K. R. et al. Environmental risk factors associated with the presence of *Mycobacterium ulcerans* in Victoria, Australia. *PLoS ONE* **17**, e0274627 (2022). 36099259 10.1371/journal.pone.0274627 PMC9469944

[9] Carson, C. et al. Potential wildlife sentinels for monitoring the endemic spread of human buruli ulcer in south-east Australia. *PLoS Negl. Trop. Dis.* **8**, e2668 (2014). 24498452 10.1371/journal.pntd.0002668 PMC3907424

[10] Fyfe, J. A. M. et al. A major role for mammals in the ecology of *Mycobacterium ulcerans*. *PLoS Negl. Trop. Dis.* **4**, e791 (2010). 20706592 10.1371/journal.pntd.0000791 PMC2919402

[11] Johnson, P. D. R. in *Buruli Ulcer: Mycobacterium ulcerans Disease* (eds Pluschke, G. & Roltgen, K.) 61–76 (Springer, 2019). 32091675

[12] O’Brien, C. R. et al. Clinical, microbiological and pathological findings of *Mycobacterium ulcerans* infection in three Australian possum species. *PLoS Negl. Trop. Dis.* **8**, e2666 (2014). 24498451 10.1371/journal.pntd.0002666 PMC3907337

[13] Xu, R. W., Stinear, T. P., Johnson, P. D. & O’Brien, D. P. Possum bites man: case of Buruli ulcer following possum bite. *Med J. Aust.* **216**, 452–453 (2022). 35445409 10.5694/mja2.51505

[14] Johnson, P. D. R. et al. *Mycobacterium ulcerans* in mosquitoes captured during outbreak of Buruli ulcer, southeastern Australia. *Emerg. Infect. Dis.* **13**, 1653–1660 (2007). 18217547 10.3201/eid1311.061369 PMC3375796

[15] Fyfe, J. A. et al. Development and application of two multiplex real-time PCR assays for the detection of *Mycobacterium ulcerans* in clinical and environmental samples. *Appl. Environ. Microbiol.* **73**, 4733–4740 (2007). 17526786 10.1128/AEM.02971-06 PMC1951036

[16] Quek, T. Y. et al. Risk factors for *Mycobacterium ulcerans* infection, southeastern Australia. *Emerg. Infect. Dis.* **13**, 1661–1666 (2007). 18217548 10.3201/eid1311.061206 PMC3375781

[17] Landier, J. et al. Adequate wound care and use of bed nets as protective factors against Buruli ulcer: results from a case control study in Cameroon. *PLoS Negl. Trop. Dis.* **5**, e1392 (2011). 22087346 10.1371/journal.pntd.0001392 PMC3210760

[18] Pouillot, R. et al. Risk factors for Buruli ulcer: a case control study in Cameroon. *PLoS Negl. Trop. Dis.* **1**, e101 (2007). 18160977 10.1371/journal.pntd.0000101 PMC2154388

[19] Lavender, C. J. et al. Risk of Buruli ulcer and detection of *Mycobacterium ulcerans* in mosquitoes in southeastern Australia. *PLoS Negl. Trop. Dis.* **5**, e1305 (2011). 21949891 10.1371/journal.pntd.0001305 PMC3176747

[20] Wallace, J. R. et al. *Mycobacterium ulcerans* low infectious dose and mechanical transmission support insect bites and puncturing injuries in the spread of Buruli ulcer. *PLoS Negl. Trop. Dis.* **11**, e0005553 (2017). 28410412 10.1371/journal.pntd.0005553 PMC5406025

[21] Australian Bureau of Meteorology. Summary statistics Mornington http://www.bom.gov.au/climate/averages/tables/cw_086079.shtml (2022).

[22] World Health Organization. Number of new reported cases of Buruli ulcer https://apps.who.int/gho/data/node.main.A1631 (2023).

[23] Mornington Peninsula Shire. Mornington Peninsula Shire population forecasts https://forecast.id.com.au/mornington-peninsula (2023).

[24] Buultjens, A. H. et al. Comparative genomics shows that mycobacterium ulcerans migration and expansion preceded the rise of Buruli ulcer in Southeastern Australia. *Appl. Environ. Microbiol.* 10.1128/AEM.02612-17 (2018). PMC5881063 29439984

[25] Taouk, M. L. et al. Characterisation of *Treponema pallidum* lineages within the contemporary syphilis outbreak in Australia: a genomic epidemiological analysis. *Lancet Microbe* **3**, e417–e426 (2022). 35659903 10.1016/S2666-5247(22)00035-0

[26] Vandelannoote, K. et al. Statistical modelling based on structured surveys of Australian native possum excreta harbouring *Mycobacterium ulcerans* predicts Buruli ulcer occurrence in humans. *eLife* 10.7554/eLife.84983 (2023). PMC10154024 37057888

[27] Endersby, N. M. et al. Evidence of cryptic genetic lineages within *Aedes notoscriptus* (Skuse). *Infect. Genet. Evol.* **18**, 191–201 (2013). 23681021 10.1016/j.meegid.2013.04.035

[28] Singh, A., McBride, W. J. H., Govan, B., Pearson, M. & Ritchie, S. A. A survey on *Mycobacterium ulcerans* in mosquitoes and march flies captured from endemic areas of northern Queensland, Australia. *PLoS Negl. Trop. Dis.* **13**, e0006745 (2019). 30789904 10.1371/journal.pntd.0006745 PMC6400404

[29] Yerramilli, A. et al. The location of Australian Buruli ulcer lesions—implications for unravelling disease transmission. *PLoS Negl. Trop. Dis.* **11**, e0005800 (2017). 28821017 10.1371/journal.pntd.0005800 PMC5584971

[30] Flies, E. J., Flies, A. S., Fricker, S. R., Weinstein, P. & Williams, C. R. Regional comparison of mosquito bloodmeals in south Australia: implications for Ross River virus ecology. *J. Med. Entomol.* **53**, 902–910, 909 (2016). 27113100 10.1093/jme/tjw035

[31] Oshaghi, M. A. et al. Effects of post-ingestion and physical conditions on PCR amplification of host blood meal DNA in mosquitoes. *Exp. Parasitol.* **112**, 232–236 (2006). 16364301 10.1016/j.exppara.2005.11.008

[32] Kay, B. H., Boyd, A. M., Ryan, P. A. & Hall, R. A. Mosquito feeding patterns and natural infection of vertebrates with Ross River and Barmah Forest viruses in Brisbane, Australia. *Am. J. Trop. Med. Hyg.* **76**, 417–423 (2007). 17360861

[33] Doig, K. D. et al. On the origin of *Mycobacterium ulcerans*, the causative agent of Buruli ulcer. *BMC Genomics* **13**, 258 (2012). 22712622 10.1186/1471-2164-13-258 PMC3434033

[34] Stinear, T. P. et al. Reductive evolution and niche adaptation inferred from the genome of *Mycobacterium ulcerans*, the causative agent of Buruli ulcer. *Genome Res.* **17**, 192–200 (2007). 17210928 10.1101/gr.5942807 PMC1781351

[35] Eisen, R. J. & Gage, K. L. Transmission of flea-borne zoonotic agents. *Annu. Rev. Entomol.* **57**, 61–82 (2012). 21888520 10.1146/annurev-ento-120710-100717

[36] Kernif, T., Leulmi, H., Raoult, D. & Parola, P. Emerging tick-borne bacterial pathogens. *Microbiol. Spectr.* 10.1128/microbiolspec.EI10-0012-2016 (2016). 27337487

[37] Ryden, P. et al. Outbreaks of tularemia in a boreal forest region depends on mosquito prevalence. *J. Infect. Dis.* **205**, 297–304 (2012). 22124130 10.1093/infdis/jir732 PMC3244368

[38] Thelaus, J. et al. *Francisella tularensis* subspecies *holarctica* occurs in Swedish mosquitoes, persists through the developmental stages of laboratory-infected mosquitoes and is transmissible during blood feeding. *Microb. Ecol.* **67**, 96–107 (2014). 24057273 10.1007/s00248-013-0285-1 PMC3907667

[39] Abdellahoum, Z., Maurin, M. & Bitam, I. Tularemia as a mosquito-borne disease. *Microorganisms*10.3390/microorganisms9010026 (2020). PMC7823759 33374861

[40] Hendry, T. A. et al. Ongoing transposon-mediated genome reduction in the luminous bacterial symbionts of deep-sea ceratioid Anglerfishes. *mBio* 10.1128/mBio.01033-18 (2018). PMC6020299 29946051

[41] Perreau, J. & Moran, N. A. Genetic innovations in animal-microbe symbioses. *Nat. Rev. Genet.* **23**, 23–39 (2022). 34389828 10.1038/s41576-021-00395-z PMC8832400

[42] Drucker, M. & Then, C. Transmission activation in non-circulative virus transmission: a general concept? *Curr. Opin. Virol.* **15**, 63–68 (2015). 26318641 10.1016/j.coviro.2015.08.006

[43] Archer, M. S. *The Ecology of Invertebrate Associations with Vertebrate Carrion in Victoria, with Reference to Forensic Entomology*. PhD thesis, Univ. of Melbourne (2002).

[44] Lang, M. D., Allen, G. R. & Horton, B. J. Blowfly succession from possum (*Trichosurus vulpecula*) carrion in a sheep-farming zone. *Med. Vet. Entomol.* **20**, 445–452 (2006). 17199756 10.1111/j.1365-2915.2006.00654.x

[45] Skopyk, A. D., Forbes, S. L. & LeBlanc, H. N. Recognizing the inherent variability in Dipteran colonization and decomposition rates of human donors in Sydney. *Aust. J. Clin. Health Sci.* **6**, 102–119 (2021).

[46] Djouaka, R. et al. Evidences of the low implication of mosquitoes in the transmission of *Mycobacterium ulcerans*, the causative agent of Buruli ulcer. *Can. J. Infect. Dis. Med. Microbiol.* **2017**, 1324310 (2017). 28932250 10.1155/2017/1324310 PMC5592421

[47] Australian Bureau of Statistics. 2016 Census QuickStats https://quickstats.censusdata.abs.gov.au/census_services/getproduct/census/2016/quickstat/SSC22187?opendocument (2021).

[48] Australian Bureau of Statistics. (Australian Bureau of Statistics, 2014).

[49] Russell, R. C. *A Colour Photo Atlas of Mosquitoes of Southeastern Australia* (Univ. of Sydney Printing Service, 1996).

[50] Dobrotworsky, N. V. *The Mosquitoes of Victoria (Diptera, Culicidae)* (Melbourne Univ. Press, 1965).

[51] Liehne, P. F. S. *An Atlas of the Mosquitoes of Western Australia* (Health Department of Western Australia, 1991).

[52] Folmer, O., Black, M., Hoeh, W., Lutz, R. & Vrijenhoek, R. DNA primers for amplification of mitochondrial cytochrome *c* oxidase subunit I from diverse metazoan invertebrates. *Mol. Mar. Biol. Biotechnol.* **3**, 294–299 (1994). 7881515

[53] PooledInfRate v.4.0: a Microsoft Office Excel add-in to compute prevalence estimates from pooled samples (Fort Collins, 2009).

[54] R Core Team. *R: A Language and Environment for Statistical Computing* (R Foundation for Statistical Computing, 2021).

[55] Illumina. Nextera Flex for enrichment with RNA probes https://support.illumina.com/content/dam/illumina-support/documents/documentation/chemistry_documentation/samplepreps_nextera/nextera-flex-enrichment/nextera-flex-for-enrichment-rna-probes-demonstrated-protocol-1000000070581-01.pdf (2023).

[56] van Buuren, S. & Groothuis-Oudshoorn, K. mice: multivariate imputation by chained equations in R. *J. Stat. Softw.* **45**, 1–67 (2011).

[57] Price, M. N., Dehal, P. S. & Arkin, A. P. FastTree: computing large minimum evolution trees with profiles instead of a distance matrix. *Mol. Biol. Evol.* **26**, 1641–1650 (2009). 19377059 10.1093/molbev/msp077 PMC2693737

[58] Revell, L. phytools: An R package for phylogenetic comparative biology (and other things). *Methods Ecol. Evol.* **3**, 217–223 (2012).

[59] Becker, R. A. & Wilks, A. R. *Constructing a Geographical Database* (1995).

[60] Buultjens, A. H. Genomic analysis of mosquito, possum and human derived *Mycobacterium ulcerans* genomes https://github.com/abuultjens/Mosquito_possum_human_genomic_analysis (2023).

[61] Townzen, J. S., Brower, A. V. Z. & Judd, D. D. Identification of mosquito bloodmeals using mitochondrial cytochrome oxidase subunit I and cytochrome b gene sequences. *Med. Vet. Entomol.* **22**, 386–93 (2008). 19120966 10.1111/j.1365-2915.2008.00760.x

[62] Song, L. & Florea, L. Rcorrector: efficient and accurate error correction for Illumina RNA-seq reads. *Gigascience*. **4**, 48 (2015). 26500767 10.1186/s13742-015-0089-y PMC4615873

[63] Grabherr, M. G. et al. Full-length transcriptome assembly from RNA-seq data without a reference genome. *Nat. Biotechnol.* **29**, 644–52 (2011). 21572440 10.1038/nbt.1883 PMC3571712

[64] Li, H. & Durbin, R. Fast and accurate long-read alignment with Burrows-Wheeler transform. *Bioinformatics.* **26**, 589–95 (2010). 20080505 10.1093/bioinformatics/btp698 PMC2828108

[65] Darriba, D., Taboada, G. L., Doallo, R. & Posada, D. jModelTest 2: more models, new heuristics and parallel computing. *Nat. Methods* **9**, 772 (2012). 22847109 10.1038/nmeth.2109 PMC4594756

[66] Guindon, S. & Gascuel, O. A simple, fast, and accurate algorithm to estimate large phylogenies by maximum likelihood. *Syst. Biol.* **52**, 696–704 (2003). 14530136 10.1080/10635150390235520

[67] QGIS Geographic Information System v.3.16.7 (Open Source Geospatial Foundation Project, 2022).

[68] Australian Bureau of Statistics. Victoria mesh blocks ASGS edition 2011 digital boundaries in ESRI shapefile https://www.abs.gov.au/AUSSTATS/abs@.nsf/DetailsPage/1270.0.55.001July%202011 (2011).

[69] Australian Bureau of Statistics. https://www.abs.gov.au (2023).

[70] Kulldorff, M. A spatial scan statistic. *Commun. Stat. Theory Methods* **26**, 1481–1496 (1997).

[71] Loftus, M. J. et al. The incubation period of Buruli ulcer (*Mycobacterium ulcerans* infection) in Victoria, Australia—remains similar despite changing geographic distribution of disease. *PLoS Negl. Trop. Dis.* **12**, e0006323 (2018). 29554096 10.1371/journal.pntd.0006323 PMC5875870

[72] Trubiano, J. A., Lavender, C. J., Fyfe, J. A., Bittmann, S. & Johnson, P. D. The incubation period of Buruli ulcer (*Mycobacterium ulcerans* infection). *PLoS Negl. Trop. Dis.* **7**, e2463 (2013). 24098820 10.1371/journal.pntd.0002463 PMC3789762

[73] Buultjens, A. H. BU-3-way-SatScan https://github.com/abuultjens/BU-3-way-SatScan (2023).

[74] Pebesma, E. Simple features for R: standardized support for spatial vector data. *R J.* **10**, 439–446 (2018).

