# Publisher Correction: Mosquitoes provide a transmission route between possums and humans for Buruli ulcer in southeastern Australia

**DOI:** 10.1038/s41564-024-01693-y

**Published:** 2024-04-15

**Authors:** Peter T. Mee, Andrew H. Buultjens, Jane Oliver, Karen Brown, Jodie C. Crowder, Jessica L. Porter, Emma C. Hobbs, Louise M. Judd, George Taiaroa, Natsuda Puttharak, Deborah A. Williamson, Kim R. Blasdell, Ee Laine Tay, Rebecca Feldman, Mutizwa Odwell Muzari, Chris Sanders, Stuart Larsen, Simon R. Crouch, Paul D. R. Johnson, John R. Wallace, David J. Price, Ary A. Hoffmann, Katherine B. Gibney, Timothy P. Stinear, Stacey E. Lynch

**Affiliations:** 1 Centre for AgriBioscience, AgriBio, Agriculture Victoria, Bundoora, Victoria Australia; 2 Department of Microbiology and Immunology, Doherty Institute for Infection and Immunity, University of Melbourne, Melbourne, Victoria Australia; 3 Department of Infectious Diseases, Doherty Institute for Infection and Immunity, University of Melbourne, Melbourne, Victoria Australia; 4 Victorian Infectious Diseases Reference Laboratory, Doherty Institute for Infection and Immunity, Melbourne Health, Melbourne, Victoria Australia; 5 Australian Centre for Disease Preparedness, CSIRO, Geelong, Victoria Australia; 6 Department of Health, Melbourne, Victoria Australia; 7 Medical Entomology, Tropical Public Health Services Cairns, Cairns and Hinterland Hospital and Health Services, Cairns, Queensland Australia; 8 South East Public Health Unit, Monash Health, Clayton, Victoria Australia; 9 North East Public Health Unit, Austin Health, Heidelberg, Victoria Australia; 10 Department of Biology, Millersville University, Millersville, PA USA; 11 Centre for Epidemiology and Biostatistics, Melbourne School of Population and Global Health, University of Melbourne, Parkville, Victoria Australia; 12 Pest and Environmental Adaptation Research Group, School of BioSciences, Bio21 Institute, University of Melbourne, Parkville, Victoria Australia; 13 WHO Collaborating Centre for Mycobacterium ulcerans, Doherty Institute for Infection and Immunity, Melbourne, Victoria Australia

**Keywords:** Bacteriology, Bacterial infection

Correction to: *Nature Microbiology* 10.1038/s41564-023-01553-1, published online 23 January 2024.

In the version of the article initially published, Extended Data Tables 1–3 were missing. They have now been added to the HTML and PDF versions of the article.

