# Supplementary material for: Mosquitoes provide a transmission route between possums and humans for Buruli ulcer in southeastern Australia
Source: Nat Microbiol. 2024 Jan 23;9(2):377–89. doi: 10.1038/s41564-023-01553-1 (PMC10847040; doi:10.1038/s41564-023-01553-1)
Supplement: Supplementary file 1 — Supplementary Figs. 1–3 and Tables 1–3. [file 41564_2023_1553_MOESM1_ESM.pdf]

nature microbiology

Article https://doi.org/10.1038/s41564-023-01553-1

Mosquitoes provide a transmission route between possums and humans for Buruli ulcer in southeastern Australia

In the format provided by the authors and unedited

List of supplementary materials:

Tables

- Supplementary Table 1| Male mosquitoes collected and screened for *M. ulcerans* on the Mornington Peninsula.
- Supplementary Table 2| *M. ulcerans* qPCR testing results for positive IS2404, IS2606 and KR in *Ae. notoscriptus, Ae. camptorhynchus* and *C. hilli*.
- Supplementary Table 3| Breakdown of the number of individual or pools of female *Ae. notoscriptus* screened each year.

Figures

- Supplementary Fig. 1| Maximum likelihood phylogenetic tree based on an 871 bp region of the COI gene.
- Supplementary Fig. 2| IS2404 qPCR synthetic positive control
- Supplementary Fig. 3| IS2404 qPCR standard curve

Datasets

- Supplementary dataset 1| Mosquito trapping details
- Supplementary dataset 2 | Accession details for *M. ulcerans* DNA sequence reads used in this study
- Supplementary dataset 3| *M. ulcerans* SNP alleles used for phylogenomic inference
- Supplementary dataset 4| Oligonucleotide sequences of RNA baits used for *M. ulcerans* genome-enrichment sequencing.

Source Data file

- Source data| Extended Fig. 2

Supplementary Table 1| Male mosquitoes collected and screened for *M. ulcerans* on the Mornington Peninsula.

Species	No. collected/No. screened (No. positive for *M. ulcerans*)					Total
	Dec 16-Apr 17	Nov 17-May 18	Dec 18-May 19	Nov 19-Mar 20	Feb 21-Mar 21	
Ae. notoscriptus		2/2	7/7	44/24	5/5	58/38
Ae. rubrithorax			1/1			1/1
Cq. linealis	9/9	24/24		67/17		100/50
Cx. australicus		11/11	2/2	303/16		316/29
Cx. globocoxitus				619/175		619/175
Cx. molestus	1/1		7/7	69/14	2/2	79/24
Cx. quinquefasciatus		32/32	1/1	108/76		141/109
Tp. tasmaniensis				3/3		3/3
Total	10/10	69/69	18/18	1,213/325	7/7	1,317/429

Supplementary Table 2| *Mycobacterium ulcerans* qPCR testing results for positive IS2404, IS2606 and KR in *Ae. notoscriptus*, *Ae. camptorhynchus* and *C. hilli*.

Species (season collected)	Location	qPCR assay			*M. ulcerans* genome equivalents/mosquito[1]
		IS2404	IS2606	KR	
Ae. notoscriptus (16/17)	Sorrento	33.64	35.18	34.94	401
Ae. notoscriptus (16/17)	Tootgarook	36.41	-	-	76
Ae. notoscriptus (17/18)	Capel Sound	38.21	-	-	26
Ae. notoscriptus (17/18)	Capel Sound	36.34	NT[2]	-	79
Ae. notoscriptus (17/18)	Capel Sound	38.27	-	-	25
Ae. notoscriptus (17/18)	Capel Sound	36.89	-	-	57
Ae. notoscriptus (17/18)	St Andrews Beach	35.02	-	-	175
Ae. notoscriptus (17/18)	Tootgarook	32.62	NT	32.18	740
Ae. notoscriptus (17/18)	Tootgarook	38.22	NT	-	25
Ae. notoscriptus (17/18)	Tootgarook	33.40	-	34.76	463
Ae. notoscriptus (18/19)	Rye	36.79	NT	32.31	60
Ae. notoscriptus (18/19)	Rye	36.67	NT	35.91	65
Ae. notoscriptus (18/19)	Rye	36.06	NT	-	93
Ae. notoscriptus (18/19)	Rye	35.56	39.371	34.95	126
Ae. notoscriptus (18/19)	Rye	36.34	NT	35.61	79
Ae. notoscriptus (18/19)	Rye	36.07	38.01	35.41	93
Ae. notoscriptus (18/19)	Rye	33.70	32.49	31.62	386
Ae. notoscriptus (18/19)	Rye	29.74	38.36	33.36	4183
Ae. notoscriptus (18/19)	Rye	31.51	38.90	33.67	1443
Ae. notoscriptus (18/19)	Rye	36.89	NT	-	57
Ae. notoscriptus (18/19)	Rye	33.32	39.00	35.92	486
Ae. notoscriptus (18/19)	Rye	37.25	-	35.80	46
Ae. notoscriptus (18/19)	Rye	35.11	-	34.19	165
Ae. notoscriptus (18/19)	Rye	36.44	40.07	35.02	74
Ae. notoscriptus (18/19)	Rye	35.27	45.00	35.49	150
Ae. notoscriptus (19/20)	Blairgowrie	35.60	37.90	33.02	123
Ae. notoscriptus (19/20)	Blairgowrie	38.56	39.89	-	21
Ae. notoscriptus (19/20)	Blairgowrie	37.45	38.58	35.23	40
Ae. notoscriptus (19/20)	Rye	38.11	39.96	38.86	27
Ae. notoscriptus (19/20)	Rye	35.14	39.55	-	163
Ae. notoscriptus (19/20)	Rye	39.15	-	-	15
Ae. notoscriptus (19/20)	Rye	33.02	35.18	32.44	582
Ae. notoscriptus (19/20)	Rye	36.76	37.03	35.15	61
Ae. notoscriptus (19/20)	Rye	37.96	38.67	33.48	30
Ae. notoscriptus (19/20)	Rye	36.09	34.62	32.86	92
Ae. notoscriptus (19/20)	Rye	37.86	39.25	39.26	32
Ae. notoscriptus (19/20)	Rye	36.51	36.79	34.07	71
Ae. notoscriptus (19/20)	Sorrento	37.73	37.08	34.86	34
Ae. notoscriptus (19/20)	Sorrento	38.51	38.80	36.34	21
Ae. notoscriptus (20/21)	Blairgowrie	33.47	34.52	29.36	489
Ae. notoscriptus (20/21)	Blairgowrie	34.15	35.05	30.10	295
Ae. notoscriptus (20/21)	Blairgowrie	38.64	40.21	35.24	20
Ae. notoscriptus (20/21)	Blairgowrie	31.20	33.26	28.52	1738
Ae. notoscriptus (20/21)	Blairgowrie	39.65	-	-	11
Ae. notoscriptus (20/21)	Rye	37.50	-	NT	39
Ae. notoscriptus (20/21)	Rye	37.48	-	-	40
Ae. camptorhynchus (19/20)	Sorrento	41.21	43.10	35.25	
C. hilli (18/19)	Rye	35.9215	34.69	-	
C. hilli (18/19)	Rye	37.541	-	38.26	

[1] Note that insect DNA was eluted in 100uL and 2uL was used for qPCR; [2]NT = Not tested

Supplementary Table 3| Breakdown of the number of individual or pools of female *Ae. notoscriptus* that were screened each year.

Year	Screening size	No. individuals or pools	Number positive
2016-17	Individuals screened	4	1
	Pools <15 (total no *Ae. notoscriptus* screened in pools 170)	23	1
2017-18	Individuals screened	367	8
	Pools <15	0	0
2018-19	Individuals screened	112	3
	Pools <15 (total no *Ae. notoscriptus* screened in pools 1667)	341	12
2019-20	Individuals screened	448	3
	Pools <15 (total no *Ae. notoscriptus* screened in pools 3882)	383	11
2021	Individuals screened	1247	7
	Pools <15	0	0

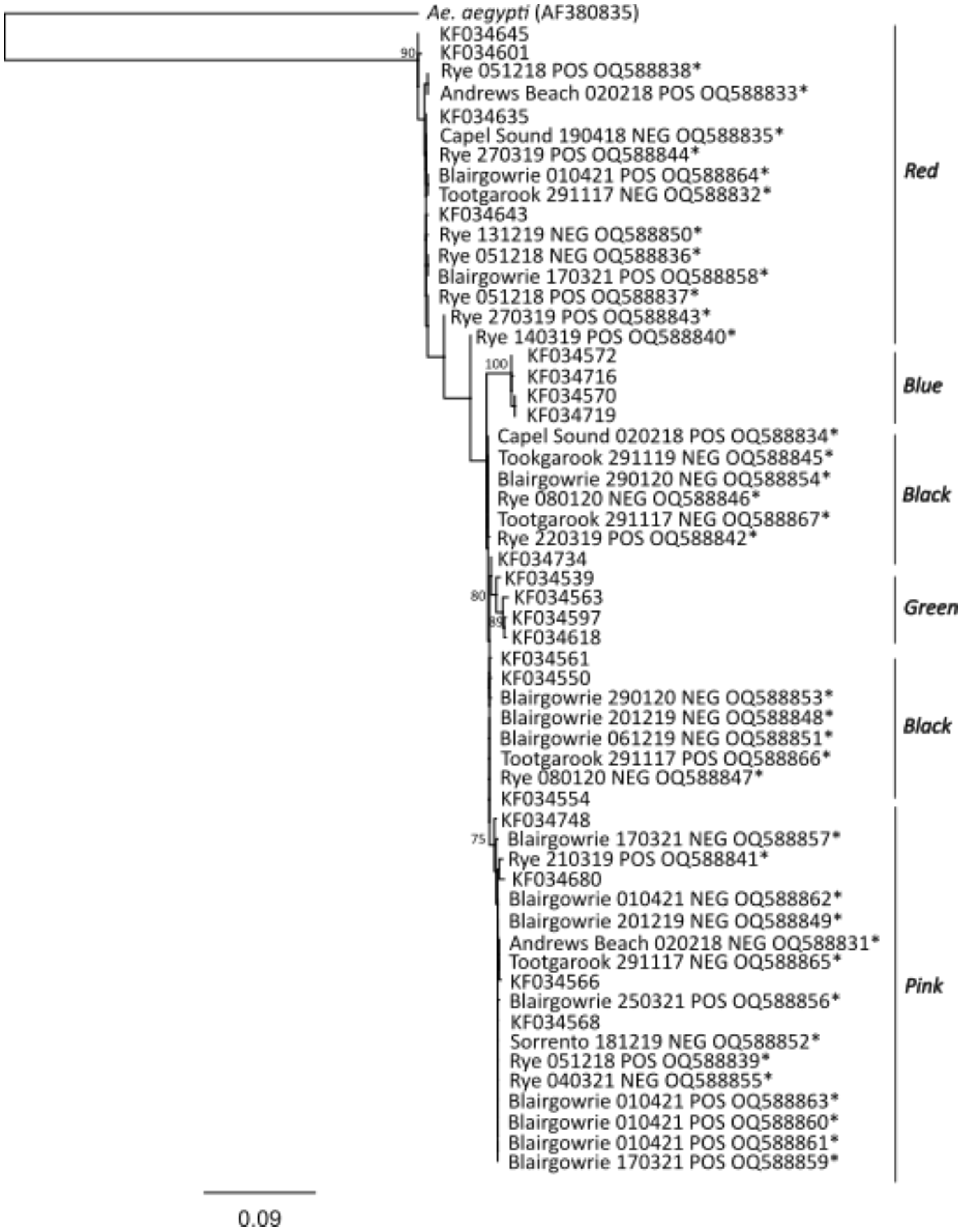

***Supplementary Fig. 1| Maximum likelihood phylogenetic tree based on an 871 bp region of the COI gene**. Labelling indicates capture location, collection date, M. ulcerans infection status and accession number. Clades are labelled based on Endersby et al. 2013, with four well-supported clades (blue, red, green and pink) and a fifth poorly supported clade (black). Insects from the blue clade had previously been collected in the subtropics of Queensland (QLD), green in the tropics of QLD, pink in temperate southern Australia, red from a broad distribution along the east coast and black from New Zealand and south-eastern Australia. The Hasegawa-Kishino-Yano (HKY) substitution model was used with 1,000 bootstrap replicates. Bootstrap proportions (BSP ≥ 70%) are indicated alongside the nodes. The number of nucleotide substitutions per site is represented by the scale bar. Aedes aegypti was used as an outgroup. * DNA Sequences generated in this study.*

A

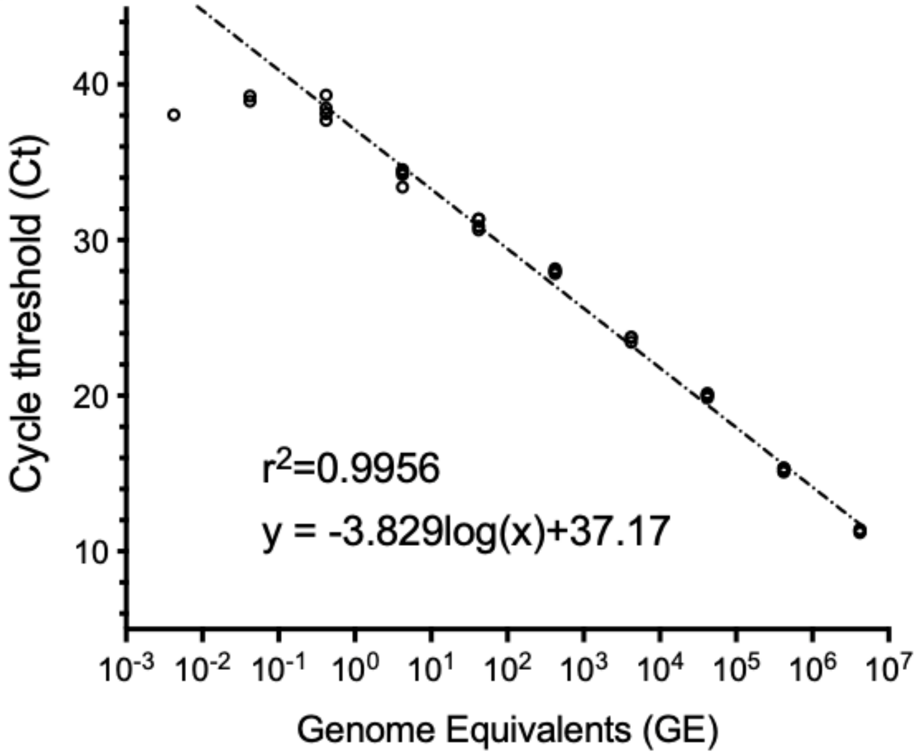

B

gDNA conc. (ng/uL)	Genome Equivalents	Rep 1	Rep 2	Rep 3	Rep 4
**12.1**	4200000	11.34	11.2	11.4	11.27
**1.2**	420000	15.09	15.17	15.35	15.35
**0.12**	42000	19.87	20.02	20.05	20.15
**0.012**	4200	23.75	23.74	23.44	-
**0.0012**	420	27.95	28.02	27.84	28.16
**0.00012**	42	30.89	31.34	31.35	30.66
**0.000012**	4.2	34.38	33.4	34.53	34.21
**0.0000012**	0.42	39.32	37.68	38.47	38.12

Supplementary Fig. 2| *IS2404 qPCR standard curve. (A) Plot of 10-fold dilution series of M. ulcerans genomic DNA tested by IS2404 Taqman real-time PCR (each dilution tested in quadruplicate). The x-axis is log_{10} scale. (B) Cycle-threshold (Ct) values for each genomic DNA dilution used to prepare the plot in (A). Results for four independent qPCR reactions are shown.*

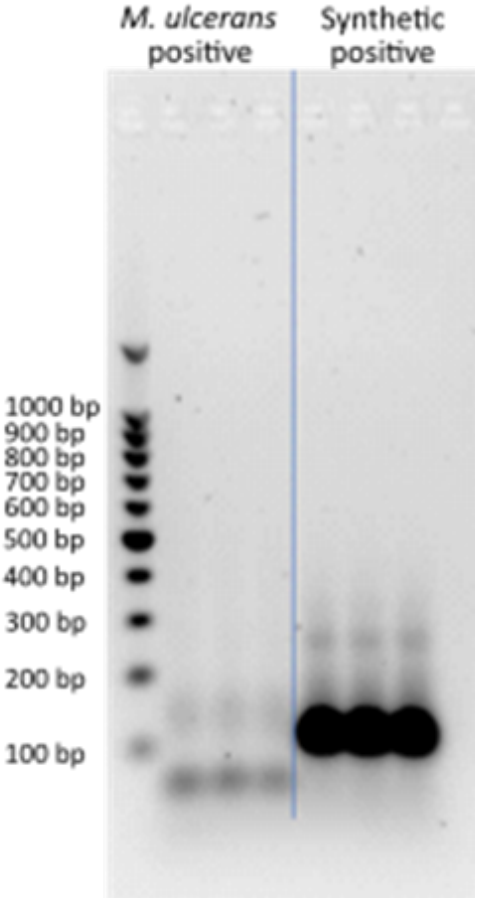

Supplementary Fig. 3| *IS2404 qPCR synthetic positive control. Comparison of the size of the qPCR amplicon (electrophoresis on 1% agarose gel) between a real M. ulcerans positive control using genomic DNA and the synthetic DNA positive control used for the screening assays.*
