# Supplementary material for: Mosquitoes provide a transmission route between possums and humans for Buruli ulcer in southeastern Australia
Source: Nat Microbiol. 2024 Jan 23;9(2):377–89. doi: 10.1038/s41564-023-01553-1 (PMC10847040; doi:10.1038/s41564-023-01553-1)
Supplement: Supplementary file 2 — Reporting Summary [file 41564_2023_1553_MOESM2_ESM.pdf]

nature portfolio

Corresponding author(s): Timothy Stinear, Peter Mee

Last updated by author(s): 18/10/2023

Reporting Summary

Nature Portfolio wishes to improve the reproducibility of the work that we publish. This form provides structure for consistency and transparency in reporting. For further information on Nature Portfolio policies, see our Editorial Policies and the Editorial Policy Checklist.

Statistics

For all statistical analyses, confirm that the following items are present in the figure legend, table legend, main text, or Methods section.

n/a	Confirmed	
☐	☒	The exact sample size (*n*) for each experimental group/condition, given as a discrete number and unit of measurement
☐	☒	A statement on whether measurements were taken from distinct samples or whether the same sample was measured repeatedly
☐	☒	The statistical test(s) used AND whether they are one- or two-sided<br>*Only common tests should be described solely by name; describe more complex techniques in the Methods section.*
☐	☒	A description of all covariates tested
☒	☐	A description of any assumptions or corrections, such as tests of normality and adjustment for multiple comparisons
☐	☒	A full description of the statistical parameters including central tendency (e.g. means) or other basic estimates (e.g. regression coefficient) AND variation (e.g. standard deviation) or associated estimates of uncertainty (e.g. confidence intervals)
☐	☒	For null hypothesis testing, the test statistic (e.g. *F*, *t*, *r*) with confidence intervals, effect sizes, degrees of freedom and *P* value noted<br>*Give P values as exact values whenever suitable.*
☒	☐	For Bayesian analysis, information on the choice of priors and Markov chain Monte Carlo settings
☐	☒	For hierarchical and complex designs, identification of the appropriate level for tests and full reporting of outcomes
☒	☐	Estimates of effect sizes (e.g. Cohen's *d*, Pearson's *r*), indicating how they were calculated

Our web collection on statistics for biologists contains articles on many of the points above.

Software and code

Policy information about availability of computer code

Data collection	Microsoft Excel (vl6.0); Atlas of Medical Entomology (Gaia Resources, V3.4.4)
Data analysis	The following software was used: Microsoft Excel (vl6.73). Geneious Prime (v2019.2.l); R (v 4.0.2); GraphPad Prism (v9.5.l); Snippy (v4.4.5); FastTree (v2.l.10); BWA-MEM v0.7.17; QGIS v.3.16.7 jModelTest2 v2.l.10; in PhyML v3.3.2; SaTScan version 10.1.0; Custom code https://github.com/abuultjens/BU-3-way-SatScan; https://github.com/abuultjens/Mosquito_possum_human_genomic_analysis

For manuscripts utilizing custom algorithms or software that are central to the research but not yet described in published literature, software must be made available to editors and reviewers. We strongly encourage code deposition in a community repository (e.g. GitHub). See the Nature Portfolio guidelines for submitting code & software for further information.

Data

Policy information about availability of data

All manuscripts must include a data availability statement. This statement should provide the following information, where applicable:

- Accession codes, unique identifiers, or web links for publicly available datasets
- A description of any restrictions on data availability
- For clinical datasets or third party data, please ensure that the statement adheres to our policy

DNA Sequencing data generated in this project are available under these NCBI GenBank accession numbers: PRJNA943595, PRJNA943595, NZ_CP085200.1, OQ600123, OQ6001234, OQ588831 - OQ58883167

Research involving human participants, their data, or biological material

Policy information about studies with human participants or human data. See also policy information about sex, gender (identity/presentation), and sexual orientation and race, ethnicity and racism.

Reporting on sex and gender	n/a
Reporting on race, ethnicity, or other socially relevant groupings	n/a
Population characteristics	n/a
Recruitment	n/a
Ethics oversight	Ethical approval for the use in this study of de-identified human BU case location, aggregated at mesh block level, was obtained from the Victorian Government Department of Health Human Ethics Committee under HREC/54166/DHHS-2019-179235(v3), 'Spatial risk map of Buruli ulcer infection in Victoria."

Note that full information on the approval of the study protocol must also be provided in the manuscript.

Field-specific reporting

Please select the one below that is the best fit for your research. If you are not sure, read the appropriate sections before making your selection.

☐ Life sciences ☐ Behavioural & social sciences ☒ Ecological, evolutionary & environmental sciences

For a reference copy of the document with all sections, see nature.com/documents/nr-reporting-summary-flat.pdf

Ecological, evolutionary & environmental sciences study design

All studies must disclose on these points even when the disclosure is negative.

Study description	Study involved systematic trapping and laboratory testing of mosquitoes and other arthropods
Research sample	The samples were predmoninantly female, adult mosquitoes (different species).
Sampling strategy	Mosquito trapping campaigns used Biogent Sentinal (BGS) traps (Biogent) that were baited with dry ice pellets to provide a source of carbon dioxide over an intended 12-hour period. GPS locations for all traps were recorded.
Data collection	Mosquitoes were collected by field research scientists (Chris Sanders, Stuart Larsen). BGS traps were set out at dusk and collected at dawn in shaded locations on the grassed edges of the roadways in the study area. GPS locations for all traps were recorded. Trapped mosquitoes were knocked down with CO2 by placing the catch-bag in dry ice before being transported back to the laboratory and kept at -20°C until processing.
Timing and spatial scale	The major mosquito survey was conducted between November 2019 and March 2020. The study area encompassed an area of 350 km2.
Data exclusions	n/a
Reproducibility	Mosquito trap sites were sampled repeatedly over the 4-month survey period
Randomization	Mosquito trap sites were distributed to ensure even spatial coverage across the study area
Blinding	The DNA extracted from mosquito and other arthropod samples were PCR screened for Mycobacterium ulcerans in a blinded process.

Did the study involve field work? ☒ Yes ☐ No

Field work, collection and transport

Field conditions	The study area is a mixture of urban and semi-rural and was originally covered in low-lying coastal vegetation, receives an average annual rainfall of 740 mm, and an elevation of 60 metres above sea-level.
Location	The study area was the Mornington Peninsula, encompassing the suburbs of Rye (population 8,416), Blairgowrie (population 2,313),Tootgarook (population 2,869) and Capel Sound (population 4,930). The study area encompasses an area of 350 km2 and is

	located 90 km south of Melbourne (37.8136°S, 144.9631 ° E), the capital city of Victoria 10. The area was originally covered in low-lying coastal vegetation, receives an average annual rainfall of 740 mm, and an elevation of 60 metres above sea-level.
Access & import/export	The study was conducted in partnership with the State Health Department and the local government authority (Mornington Peninsula Shire Council). No specific permit was needed to undertake the mosquito surveillance.
Disturbance	No disturbance was caused.

Reporting for specific materials, systems and methods

We require information from authors about some types of materials, experimental systems and methods used in many studies. Here, indicate whether each material, system or method listed is relevant to your study. If you are not sure if a list item applies to your research, read the appropriate section before selecting a response.

Materials & experimental systems

n/a	Involved in the study	
☒	☐	Antibodies
☒	☐	Eukaryotic cell lines
☒	☐	Palaeontology and archaeology
☐	☒	Animals and other organisms
☒	☐	Clinical data
☒	☐	Dual use research of concern
☒	☐	Plants

Methods

n/a	Involved in the study	
☒	☐	ChIP-seq
☒	☐	Flow cytometry
☒	☐	MRI-based neuroimaging

Animals and other research organisms

Policy information about studies involving animals; ARRIVE guidelines recommended for reporting animal research, and Sex and Gender in Research

Laboratory animals	n/a
Wild animals	Mosquito trapping campaigns used Biogent Sentinal (BGS) traps (Biogent) that were baited with dry ice pellets to provide a source of carbon dioxide over an intended 12-hour period. GPS locations for all traps were recorded. Other arthropod surveys used sticky traps.
Reporting on sex	Mosquitoes trapped were speciated and sexed.
Field-collected samples	Mosquitoes were frozen at -20oC upon collection and then stored at that temperature until laboratory testing.
Ethics oversight	No ethics approvals were required for arthropod trapping

Note that full information on the approval of the study protocol must also be provided in the manuscript.

Plants

Seed stocks	n/a
Novel plant genotypes	n/a
Authentication	n/a
